# A State‐of‐the‐Art Review on the Recent Advances in Exosomes in Oncogenic Virus

**DOI:** 10.1002/hsr2.70196

**Published:** 2024-11-18

**Authors:** Fatemeh Ebrahimi, Ali Modaresi Movahedi, Mohammad Sabbaghian, Vahdat Poortahmasebi

**Affiliations:** ^1^ Department of Bacteriology and Virology Faculty of Medical Sciences, Tabriz University of Medical Sciences Tabriz Iran; ^2^ Department of Medical Parasitology and Mycology Faculty of Medical Sciences, Shahid Sadoughi University of Medical Sciences Yazd Iran

**Keywords:** exosome, extracellular vesicles, miRNAs, oncovirus

## Abstract

**Background and Aims:**

Oncogenic viruses are responsible for approximately 12% of human malignancies, influencing various cancer processes through intricate interactions with host cells. Exosomes (EXOs), nanometric‐sized microvesicles involved in cell communication, have emerged as critical mediators in these interactions. This review aims to explore the mechanisms by which EXOs produced by cells infected with oncogenic viruses promote cancer growth, enhance viral transmissibility, and act as immunomodulators.

**Methods:**

A comprehensive review was conducted, focusing on recent studies highlighting the mechanisms by which EXOs facilitate the oncogenic potential of viruses. The analysis included the characterization of exosomal content, such as microRNAs (miRNAs) and proteins, and their effects on tumor microenvironments and immune responses. A search was performed using databases including PubMed, ScienceDirect, and Google Scholar. MeSH keywords related to EXOs, oncogenic viruses, and cancer were used to retrieve relevant review, systematic, and research articles.

**Results:**

Findings indicate that EXOs from oncogenic virus‐infected cells carry viral components that facilitate infection and inflammation. These EXOs alter the tumor microenvironment, contributing to the development of virus‐associated cancers. Additionally, the review highlights the growing interest among researchers regarding the implications of EXOs in cancer progression and their potential role in enhancing the oncogenicity of viruses.

**Conclusion:**

The findings underscore the pivotal role of EXOs in mediating the oncogenic effects of viruses, suggesting that targeting exosomal pathways may provide new therapeutic avenues for managing virus‐associated cancers. Further research is needed to fully elucidate the functional mechanisms of EXOs in viral oncogenesis.

## 1 Introduction

The small vesicles attached to the membrane secreted by the cells are called exosomes [1, 2]. Extracellular vesicles (EVs) can be divided into three principal groups: exosomes, microvesicles, and apoptotic bodies. Exosomes are a subpopulation of EVs originating from multivesicular bodies (MVBs), a late endosome type. Most cell types can produce these vesicles and are detectable in blood, urine, and other body fluids. Exosomes secreted in the extracellular space mediate cell‐to‐cell communication by transferring biomacromolecules, functional proteins, and nucleic acids between cells. Evidence suggests that exosomes can transfer genetic materials, including mRNAs, microRNAs (miRNAs), and other noncoding RNAs, between cells, which can modulate gene expression and cellular functions in recipient cells [2, 3]. These EVs carry many molecules involved in cell‐to‐cell communication [4]. EVs have a limited capacity to carry cargo. However, they take different types of lipids, proteins, and RNAs and transfer them from donor cells to recipient cells, affecting the function of these cells [5]. It has also been observed that nucleic acids such as RNA, miRNA, long noncoding RNAs (lncRNAs), and DNA inside the exosome are protected from degradation by enzymes in the exosome membrane [6, 7]. Viruses use exosomes to deliver RNA or DNA. These exosomes ultimately trigger the innate immune system response. They can interfere with regular cellular function and lead to disease [8]. Cancer‐causing viruses, known as oncoviruses, are responsible for approximately 20% of all human oncogenesis [9]. Exosomes can play an essential role in viral pathogenesis, including their role in human T‐cell lymphotropic virus type 1 (HTLV‐1), which can cause T‐cell lymphoma in adults [10, 11], chronic infection with hepatitis C virus (HCV), which can cause liver cancer [12, 13], human immunodeficiency virus type 1 (HIV‐1) infection, which indirectly causes cancer by suppressing the immune system [14, 15], human papillomavirus (HPV), which causes cervical cancer (CC) [16], and Epstein–Barr virus (EBV), which can lead to Burkitt's lymphoma [17, 18].

Some of these exosomes can intensify tumor growth and spread [19]. For example, in EBV‐infected nasopharyngeal carcinoma (NPC) cells, exosomes contain hypoxia‐inducible factor 1a (HIF1a) and latent membrane protein 1 (LMP1), which are involved in tumor development and progression in NPC [20, 21]. It has also been found that the exosomes associated with HPV contain miRNAs related to CC [22]. Also, studies have mentioned the presence of viral RNA, such as HCV RNA, in exosomes [23]. Ramakrishnaiah's study clarified the existence of an exosomal pathway for HCV transfer between hepatocytes [24]. A survey conducted on several cell lines found that HTLV‐1 EVs are not infectious, but these EVs can increase cell‐to‐infected cell contact [25]. About the HIV‐1 virus, extensive studies have shown that exosomes are very similar to HIV particles in many ways, such as physical properties and composition [26, 27]. According to the studies that have been done so far, it has been determined that exosome cargoes change considerably during viral infections, and many studies have shown that exosomes that are separated from virus‐infected cells contain pathogenic factors such as HCV RNA and HIV‐1 [28, 29].

Exosomes can have a fundamental activity in the infection and spread of viruses [30]. The main part of this article concerns current knowledge about the mechanisms used by oncoviruses to use exosomes and how exosomes derived from oncoviruses work. Moreover, our review often focuses on exosomes due to abundant research in this area; the principles discussed can usually be applied to EVs more broadly.

## 2 Biogenesis of Exosomes

**Figure 1:**
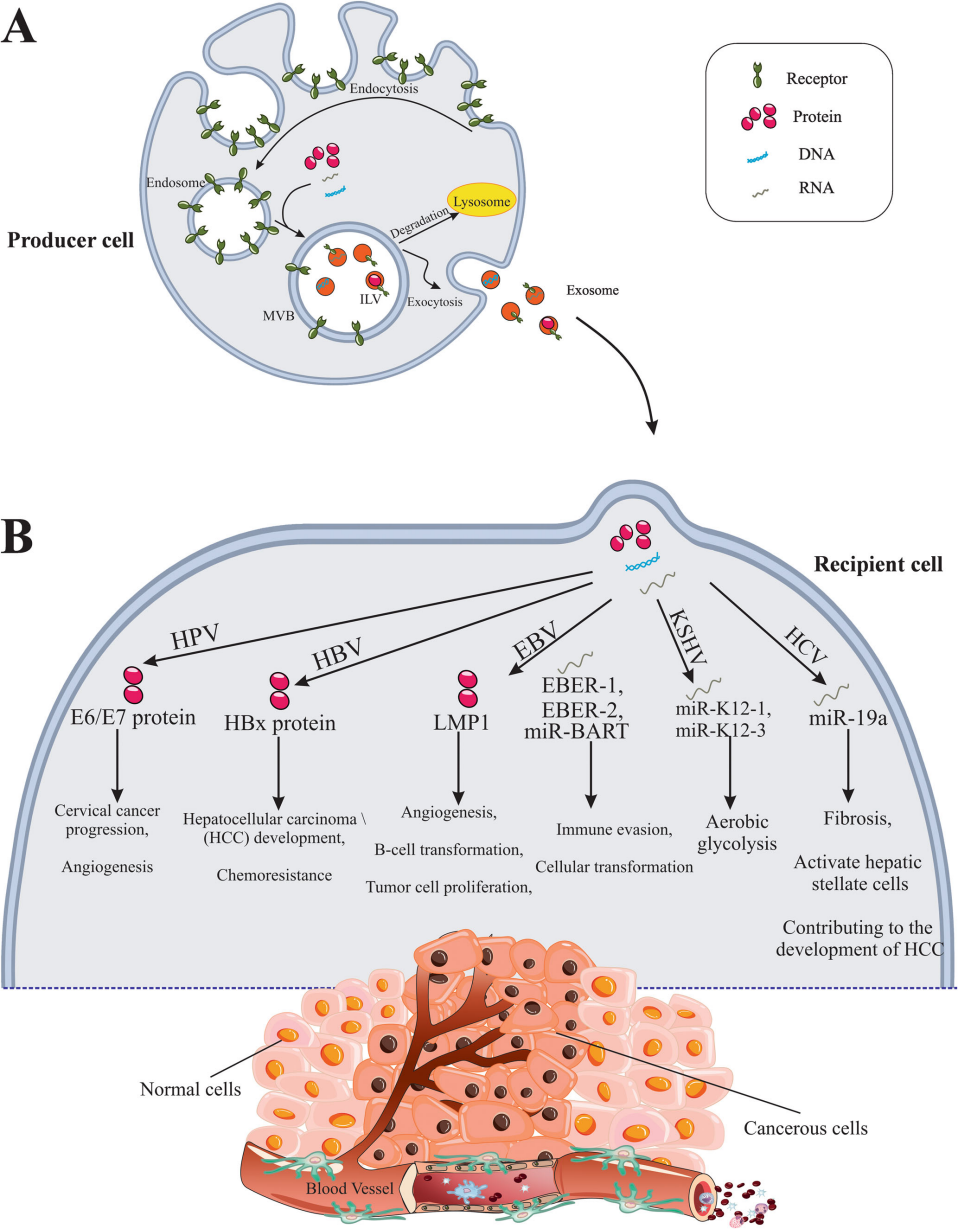
Biogenesis and the role of some exosomes in viral tumorigenesis. (A) The stages of exosome biogenesis involve complex processes such as endocytosis, formation of multivesicular bodies (MVBs), and exocytosis. This begins with the internalization of the plasma membrane and the formation of intraluminal vesicles (ILVs) within MVBs. ILVs are generated during endosomal maturation through both ESCRT‐dependent and ESCRT‐independent mechanisms. Finally, after MVBs fuse with the plasma membrane, exosomes are released into the extracellular space, playing a crucial role in intercellular communication. (B) The diverse functions of exosomes in the context of oncogenic viral infections and cancer progression.

Exosomes are called membrane‐enclosed vesicles that carry nucleic acids, proteins, and lipids and have a diameter of 30–100 nm [31]. Endosomes create exosomes via an intricate internal biosynthetic process that includes double plasma membrane (PM) invagination and the development of MVBs brimming with intraluminal vesicles (ILVs) [32, 33]. ILVs arise during endosomal maturation by ESCRT‐dependent (endosomal sorting complexes required for transmission) and non‐ESCRT‐dependent mechanisms [34]. Membrane internalization into early endosomes and subsequent migration to MVB are the first steps in the production of exosomes. ILVs are then released into the extracellular environment as exosomes after MVBs have fused with the PM. Additionally, they may join with lysosomes or autophagosomes for degradation [35]. Exosome surface proteins include tetraspanin proteins CD9, CD63, CD81, and CD82, adhesion molecules, and immunological regulatory molecules of major histocompatibility complex (MHC) classes I and II [36]. Twenty proteins, collectively called ESCRT‐0, ESCRT‐I, ESCRT‐II, and ESCRT‐III, make up ESCRT, which mediates the biogenesis process [34]. The significant abundance of Rab GTPases in isolated exosomes their involvement in exosome production. Rab GTPases are a recognized family of conserved proteins that control vesicular trafficking and membrane fusion processes [37]. For example, Rab27A and Rab27B deliver exosomes to the PM and promote their fusion with the membrane [38]. The HSP70 surface protein receptor is necessary for detecting and providing exosomes to recipient cells. As a result, the synthesis of exosomes is a complicated and continuous process that can be either ESCRT‐dependent or ESCRT‐independent. Viruses have been shown in several studies to interfere with the ESCRT pathway and affect how exosomes are secreted [39]. For instance, exosome production was higher in cells that expressed the EBV LMP1 protein than in cells that did not [40]. Similarly, the HPV E6/E7 protein also influenced the release of exosomes by HPV‐positive cancer cells [41]. The stages of exosome biogenesis are summarized in Figure 1A and Table 1.

**Table 1:** Biogenesis of exosomes.

1	Endocytosis	The plasma membrane folds inward, forming early endosomes	[31, 33]
2	Endosomal maturation	Endosomes mature into multivesicular bodies (MVBs)	[33]
3	ILV formation	Intraluminal vesicles (ILVs) form inside MVBs	[33]
4	Exosome release	Combining MVBs with the plasma membrane causes the release of ILVs in exosomes.	[34]

## 3 Mechanisms of Viral Component Transmission via Exosomes

### 3.1 Hijacking Exosome Biogenesis Pathway

Viruses proliferate by taking over and taking advantage of biological replication machinery; most effective viruses eventually destroy the host cell. Some viruses can hijack the exosome biogenesis pathway through a sequence of complexes known as ESCRT and package themselves into exosomes [42]. By concealing viral genomes, subverting the immune system, and enhancing viral infection in uncontaminated cells, viral antigens in exosomes promote viral persistence and facilitate their spread to neighboring cells without the direct interaction between viral ligands and host cell receptors [43, 44, 45]. Numerous viruses enter cells via the endocytic pathway. Endocytosis‐transmitted viruses such as HCV, Zika virus (ZV), West Nile virus (WNV), and Dengue virus (DENV) can hijack and exploit exosomal pathways for their advantage [46]. Following the “back‐fusion” of ILVs, these infectious viruses oppose late endosomes, culminating in discarding the viral genome inside the cytoplasm [42, 47]. Regarding HCV, the virus's genome may persist in ILVs and be released into exosomes, which may function as infectious particles [48]. There are numerous budding mechanisms for virions' release. Various enveloped viruses are pushed to the PM and hijack the ESCRT pathway by mimicking interactions among cellular adaptors and ESCRT components [49]. Additionally, virions penetrate the MVB, and either cause lysosomal degradation or hijack exosomal and autophagosomal pathways. Rather than hijacking the ESCRT pathway, other viruses have been identified to employ Rab GTPase complexes for replication and release. Rab5B is hijacked by hepatitis B virus (HBV) large hepatitis B surface proteins (LHBs), which causes them to be transported from the endoplasmic reticulum to the MVBs. LHBs attract HBV capsid, TSG101, and Bud within MVBs [50]. After that, MVB maturation and fusion with the PM are enhanced by HBVs' hijacking of Rab7a and Rab27 [51]. It has been demonstrated that HIV1‐proteins interact with Rab27a to stimulate the formation of exosomes [52]. The hepatitis A virus (HAV) capsid recruits ESCRT‐III, vacuolar protein sorting‐associated protein 4 (VPS4), and ALIX, enabling the viral capsid to inwardly bud inside MVBs and subsequently discharge within EVs [53]. Before HEV‐EV Rab27a‐dependent secretion, the HEV protein pORF3 interacts with TSG101 to attract the viral capsid and package it into MVBs [54, 55]. Through interaction with TSG101 and permitted absorption within MVBs, the nonstructural viral protein NS3 mediates the packaging of the bluetongue virus into EVs [56, 57].

### 3.2 Fusion With Recipient Cells

EVs play a major part in the spread of viruses and the infectiousness of healthy cells. Exosomes carrying viral particles or genetic material can fuse with recipient cells, directly spreading the virus and causing infection in new cells [58]. This can offer a cloak to both enveloped and non‐enveloped viruses, including human cytomegalovirus (CMV), human herpesvirus 6 (HHV‐6), SARS‐CoV‐2, DENV, HBV, HAV, hepatitis E virus (HEV), enterovirus 71 (EV71), and bluetongue virus, hiding them from immune monitoring while permitting viral propagation. For example, DV‐EVs carrying virus‐like particles transmit viruses to noninfected mosquito cells [59]. Furthermore, virus‐EVs transfer viral genomes that can cause active infection of recipient cells. The EV‐like particles seen in the sera of individuals infected with human pegivirus carry the pegivirus RNA genome to recipient T cells, B cells, NK cells, and monocytes, where they facilitate the viral RNA's active replication [60]. Several viruses modify EVs' lipid and protein makeup to improve binding and absorption into new cells. Virus‐EVs can exhibit phosphatidylserine (PS) on their surface, enhancing their reception into recipient cells EBV [61], HAV [62], HIV [63], ZV [64], poliovirus, rhinovirus, and coxsackievirus B3 [65]. When an infection is latent, recipient dendritic cells (DCs) absorb EBV‐EVs harboring viral miRNA because PS binds to the T cell immunoglobulin mucin domain‐1 (TIM‐1) receptor on DCs [66].

### 3.3 Immune Modulation

Exosomes can carry viral components that modulate the immune response by suppressing antiviral immune mechanisms or creating an environment conducive to viral replication. Certain virus‐EVs have host miRNAs that cause recipient cells to become less responsive to interferon (IFN) signaling, thereby increasing the likelihood of viral infection [67]. For instance, the viral nucleocapsid is transported by Ebola virus‐EVs, which affects the target cells' IFN‐1 response [68]. Viral modifications to the EV biosynthesis pathways are another way viruses have evolved to elude the innate immune responses. Autophagolysosome destruction is a barrier that coxsackievirus, rhinovirus, poliovirus, and DV have learned to avoid by concealing in autophagosome‐derived EVs [65, 69]. Likewise, several viruses can evade phagocytic degradation. For example, HIV‐1 is capable of entering MVB after immature DCs have trapped it, and then it is trafficked within EVs rather than being broken down. These EVs are linked to HIV‐1 particles and contain elevated levels of HLA‐CD9, CD63, and DR1. Through the HIV‐1 GP120 receptor, these particles cause recipient CD4 T cells to become actively infected [70]. Virus‐EVs transport viral proteins that decrease immune recognition by presenting antigens. The viral envelope glycoprotein B (gB) is present in HSV‐1‐EVs and inhibits the expression of major histocompatibility complex class II (MHC‐II) and increases MHC‐II uptake and secretion in EVs. This decreases the capacity of recipient cells to express viral antigens and trigger immune responses [71]. Antibodies can be neutralized by infectious substances transported through EVs to evade detection. For instance, neutralizing IgG antibodies only identify a portion of HCV‐EVs that carry viral RNA and proteins [24]. At last, certain virus‐EVs that carry viral proteins allow immune cells to undergo apoptosis or active infection. HBV is a hepatic cell tropism, but HBV‐EVs cause an active infection of the recipient NK cells, suppressing the antiviral immune response and promoting persistent infection [72]. In the end, interactions between viruses and EVs encourage the spread of viruses by impeding a range of innate and adaptive immune responses. These interactions disrupt the antiviral immune response and have broader tissue effects. Another recognized instance is the DNA virus known as EBV, which uses vesicular production to prevent the antiviral response. Viral proteins, such as LMP1, a pro‐oncogenic protein that promotes EBV‐infected B lymphocyte transformation and immortalization and modulates the global immune system, are released by EBV‐infected cells in the form of vesicles [73]. EVs containing LMP1 that are released by B cells limit the growth of T and NK cells, which lowers the immunological response against the virus [74].

### 3.4 Gene Expression Regulation

Exosomes can contain viral miRNAs or mRNAs that can be delivered to recipient cells, altering gene expression to favor viral replication and spread. EVs containing miRNAs from viral infections may be helpful indicators of disease states and important intermediaries of some biological processes involving cell‐to‐cell communication [75]. The EBV genome encodes a collection of miRNAs crucial for controlling different stages of viral pathogenesis, including latency and lytic reactivation. These miRNAs are essentially grouped into three primary clusters: BHRF1, BART cluster 1, and BART cluster 2 [76]. EBV‐infected B cells could also release EVs containing viral miRNAs, which modulated other cell types' responses in paracrine manner [77]. Lin et al. found that members of the miR‐200 family, generated by oral epithelial cells and transported inside EVs, were responsible for driving EBV lytic reactivation in B‐lymphocytes. Their suggested model states that the epithelium EV cargo stimulates B cells to initiate the lytic cascade again. This permits EBV reactivation and epithelial infection, where the virus replicates rapidly to enable salivary transmission [78]. The primary oncogenic proteins of HPV, E6, and E7, are known to modify the host cell cycle, promoting its malignant transformation by altering the tumor suppressors p53 and pRB, respectively [79]. Unsurprisingly, their actions involve modifications to the host miRNA repertoire that promote the overexpression of protumorigenic miRNAs like miR‐21 [80]. Proteins E6 and E7 may be artificially expressed alone to induce profound alterations in the miRNA profile in both the cell and the EV content, demonstrating their critical function in manipulating host defenses to create an environment conducive to viral establishment [81]. Circulating miRNAs may potentially be a useful biomarker for HBV infection. The hepatitis B surface antigen (HBsAg) particles can carry miRNAs linked to antigenic status and treatment response, but some miRNAs, like miR‐192‐5p, miR‐193b‐3p, and miR‐194‐5p, can be found within EVs and are thought to be useful indicators of the disease [82]. Enomoto et al. emphasized the role of miR‐192 and four additional exosomal miRNAs (miR‐21, miR‐215, miR‐221, and miR‐222) in suppressing the synthesis of IL‐21, a cytokine generated from T cells that has anti‐HBV activity [83]. In addition to using the host's miRNA to its advantage, HIV codes for a unique collection of miRNAs in its genome, most of which are members of the transactivation response (TAR) elements family [84]. EVs from HIV‐infected T cell lines included RNases Drosha and Dicer in addition to TAR miRNAs, indicating that TAR miRNAs may mature once they reach a naïve cell [85]. Interestingly, TAR miRNAs enhance viral infection in targeted cells by reducing the production of the proapoptotic protein Bim, which counteracts apoptosis in those cells [86]. Furthermore, by activating TLR3, TAR miRNAs may cause the generation of inflammatory cytokines, including IL‐6 and TNF‐β, which would contribute to HIV‐induced inflammation [87]. Host components, including miR‐122 and the proteins argonaute‐2 (Ago2) and heat shock protein 90 (HPS90), are carried by EVs produced from HCV‐infected hepatocytes, and they aid in stabilizing the viral RNA and enhancing its infectivity [88]. EVs can carry and release miRNA‐192, which alters the phenotype of the cells by increasing their synthesis of transforming growth factor β (TGF‐β). This cytokine functions as a potent stimulator of hepatic stellate cells (HSCs) and is implicated in maintaining fibrogenesis in liver tissue [89]. Moreover, it has been discovered that other factors, such as Ago2, miR‐122, and HSP90, promote HCV replication [90].

### 3.5 Creating a Favorable Environment

Exosomes can carry host cell factors that the virus has altered to create an environment more favorable for viral replication and persistence. EVs appear to be used both directly and indirectly by HIV and HCV. They can directly hijack the cellular system by rerouting cellular proteins and nucleic acids into EVs to establish an environment conducive to their replication and dissemination. They indirectly impart distinct viral components to exosomes and other vesicles, thus promoting viral pathogenesis [91]. HIV‐EVs carry the metalloprotease ADAM17, implicated in the inflammatory response, and the viral co‐receptors CCR5 and CXCR4 to other cells, expanding the virus's pool of target cells. In contrast, HCV‐EVs are packed with Ago2 and miRNAs [88]. For instance, HIV‐TAR RNA generates miRNAs that have antiapoptotic properties, promoting the survival of infected cells [92]. On the other hand, HCV‐Ago2‐miRNA122 EVs and HIV‐Nef EVs function in target cells by promoting viral replication [93]. Certain viruses regulate the packing of host components. This happens when it comes to the Kaposi's sarcoma‐associated herpesvirus (KSHV/HHV‐8). Proteins that impact the immune system, such as cleaved versions of IL‐1 and IFI16, and metabolic proteins like lactate dehydrogenase are abundant in EVs secreted by KSHV‐infected cells. As a result, KSHV‐EVs modify recipient cells' metabolism and innate immune response, which promotes viral persistence [94]. Viral and cellular proteins delivered by EVs have a variety of consequences on the target cells. Numerous of these effects permit the maintenance of cell activation, which favors viral entrance and replication and frequently advances pathogenesis.

### 3.6 Facilitation of Viral Entry

Exosomes can enhance viral entry by carrying receptors or adhesion molecules that facilitate virus binding and entering recipient cells. Microparticles transmit the HIV‐1 viral receptor CCR5 to endothelial cells that do not naturally express CCR5 and peripheral blood mononuclear cells (PBMCs), enabling HIV‐1 endothelium infection [95]. EVs transport tetraspanin CD9 and the SARS‐CoV‐2 viral receptor ACE2 across noninfected endothelium cells. CD9 promotes ACE2 aggregation at recipient cells' cell surfaces, increasing those cells' susceptibility to SARS‐CoV‐2 infection [96].

Moreover, small viruses, like hepatitis viruses (55–65 nm), can hide from the immune system by encapsulating themselves in exosomes. Since exosomes are naturally occurring vesicles, immune cells may not detect them as foreign. The encapsulated virus particles can enter cells more effectively when exosomes merge with the membranes of recipient cells [97]. Remarkably, recent research revealed that replication‐competent double‐stranded HCV RNAs are carried in significant amounts by HCV‐related exosomes. These RNAs can evade hepatocyte‐specific toll‐like receptor‐3 (TLR‐3)‐mediated innate responses, guaranteeing viral proliferation [98]. The HEV uses the cellular exosomal route to release itself through MVBs, which circulate in the blood while the virus is wrapped in protective membranes. The EVs that contain HEV are just as contagious as the virus itself [99].

All cells, even malignant and virus‐infected ones, release EVs. Moreover, serum, plasma, semen, urine, saliva, cerebrospinal fluid, amniotic fluid, bronchial alveolar lavage, breast milk, and ascites are just a few examples of the normal and pathological bodily fluids in which EVs have been found in vivo. This suggests that EVs may reach any anatomic location or space within the human body. In the context of viral infections, the biological activity of EVs might impact it in two opposite ways. EVs can influence recipient cells by stimulating viral replication or inhibiting it by inducing host immune responses.

## 4 Virus‐Associated Cancer and Exosomes

Exosomes can be secreted from different cells, such as healthy, cancerous, and virus‐infected cells [100, 101]. Studies have shown that the content of exosomes can be changed by oncogenic viruses, causing persistent infection and pathogenesis [102]. Viruses are similar to exosomes in several ways, such as their diameter, between 30 and 300 nm, and their release from the multivesicular pathway [26]. Cells infected with cancer and viruses use exosomes for different purposes, such as creating changes in their microenvironments and directing changes in the growth stages of adjacent cells. This is achieved through intercellular transfer mediated by cell signaling molecules and viral miRNAs [103]. Viruses use various strategies to escape from the immune system, survive, and reproduce, with exosomes playing a crucial role in this process [43]. The role of exosomes in mediating oncogenic viral infections is illustrated in Figure 1B.

### 4.1 Exosomes and EBV

EBV is a member of the herpesvirus family and belongs to the genus of gammaherpesviruses [104]. Infections caused by this virus are prevalent and can cause lifelong infections in B lymphocytes and epithelial cells following the initial infection [105]. The life cycle of EBV has 2 phases, which include a latent phase and a lytic phase, present in all herpes viruses. Lytic infection occurs after virus entry and replication, and replication‐related genes are activated at this stage. In the latent infection phase, the virus does not multiply but hides inside a series of cells, and in this phase, genes are expressed that keep the virus in the latent phase. Latent phase proteins include latent membrane proteins (LMP), Ebna, and short RNAs encoded by the Epstein–Barr virus (EBER) [106, 107]. Evidence has shown that exosomes produced by EBV‐infected cells are eventually internalized and can deliver viral factors to recipient cells, such as EBV‐encoded LMP and miRNAs [104, 108]. Exosomes carrying viral proteins or nucleic acids are constantly released by EBV‐infected tumor cells [109]. Studies conducted to date on exosomes derived from EBV+ tumors show that the oncogenic molecules encoded by EBV, in addition to having tumorigenic effects on noninfected cells through delivery from exosomes, can have potential immunosuppressive effects [110, 111, 112]. Previous studies have shown how exosomes produced by viruses affect how cancer develops and spreads when EBV infections are present. Viral miRNA contributes significantly to EBV's pathophysiology and life cycle by influencing cell division, transformation, and immune response invasion. EBV virus takes advantage of the host cell's miRNA mechanism to escape and adapt to immune system recognition [113]. EBV‐encoded miRNA found in exosomes can induce the secretion of T‐cell inhibitory exosomes during infection. Among the proteins involved in many cases of EBV‐related cancers are the LMPs. LMPs come in two different varieties: LMP1 and LMP2, which comprise LMP2A and LMP2B. Each oncogenic protein plays a specific role in human malignancies caused by EBV [114, 115, 116]. Exosomal LMPs have been shown to induce epithelial–mesenchymal transition (EMT) by altering the environment around tumors. They also transform B lymphocytes and ultimately promote cancer cell proliferation, migration, and invasion, especially in HL and NPC [48, 50, 54]. It has previously been demonstrated that EBV‐infected cells, particularly NPC cells, release the LMPs‐carrying exosomes [20, 21, 104]. According to recent research, LMP‐1 generated from exosomes is essential for both the immunological response to EBV infection and the growth and progression of EBV‐associated malignancies [104, 117, 118]. By producing exosomes containing LMP1 and galectin nine from EBV‐infected LCL and NPC cells, the immune system is altered through a decrease in the intensity of T‐cell activation and an increase in the killing power of natural killer cells. As a result, tumor cells can bypass the immune system, accelerating the development and spread of malignancy [119]. It has also been shown that exosomes carrying LMP1 and HIF cause tumor invasion in NPC [20]. Encapsulated LMP1 influences protein selection and transports signaling proteins into cells that are not infected. For instance, to support the invasive capacity of nasopharyngeal carcinoma, hypoxia‐inducible factor 1 was delivered into nasopharyngeal cancer cells via exosomes expressing LMP1 [20]. Exosomes contain the biological proteins epidermal growth factor receptor (EGFR) and phosphoinositide 3‐kinase (PI3K), which, when activated by protein kinase B (AKT) and extracellular signal‐regulated kinase (ERK) signaling pathways, cause nasopharyngeal cancer cells to undergo carcinogenesis [103]. Furthermore, it was noted that LMP1 not only encouraged the expression of fibroblast growth factor 2 (FGF‐2), a strong angiogenic factor involved in tumor development and spread in epithelial cells, but moreover caused FGF‐2 release through exosomes [120]. Research has shown that tetraspanin CD63, a typical exosome marker, interacts with exosomal LMP1. Through this connection, the former can avoid being degraded by lysosomes. This might increase exosomal LMP1's capacity to cause cancer. In addition, regulating exosome secretion is also one of the functions of LMP‐1, which may be related to EBV‐derived malignancies. These results show that exosomes with LMP can contribute to EBV pathogenicity through different pathways. Suppression of antiviral immune responses and promotion of varying tumor signaling pathways are among the paths they use [121]. Exosomes linked with EBV may also include EBERs and LMPs. There is widespread expression of noncoding RNAs known as EBERs (EBER‐1 and EBER‐2) by exosomal release in EBV‐infected cells [122, 123]. These EBERs significantly impact cellular transformation and innate immunity against viruses [124]. EBERs were shown to be ejected in the form of exosomes by Ahmed and colleagues, along with the EBER‐binding protein lupus antigen (La) ribonucleoprotein [122]. More research should be done to better understand the pathogenic effects of EBER‐carrying exosomes after being internalized into neighboring cells. Other viral mRNAs and miRNAs known to have a role in EBV pathogenesis and persistence may also be present in EBV exosomes [77, 125, 126, 127]. For example, DCs receiving exosomes from EBV‐transformed LCL might receive mature EBV‐encoded miRNAs to decrease the expression of EBV target genes in the cells [77]. Although the role of exosomes containing EBV in the pathogenesis of these viruses is prominent, the mechanism of their internalization in target cells and the phenotypic modification of those cells by exosomes remains unknown. The mechanism of internalization of exosomes from EBV type I and III infected cells into non‐EBV infected epithelial target cells is via caveolae‐dependent endocytosis, as described by Nanbo et al. [104]. Exosomes harboring EBV could connect to various proteins and receptors on target cells, like CD21 on B cells [128]. It is envisaged that receptor‐associated pathways will be used to mediate exosome internalization. The role of the exosome internalization pathway in the pathogenicity and stability of EBV, as well as the carcinogenesis of this virus, still needs more studies. A screening of viral proteins was conducted to find the viral proteins responsible for the exosome‐mediated increase of EBV infection. Exosomes containing the BGLF2 protein were found and discharged in EBV‐infected cells. Shortly after infection, the togoman protein BGLF2 is released into the cytoplasm from its location in the region between the nucleocapsid and the envelope. The expression of viral genes is increased, and innate immunity is suppressed by exosomes that contain the BGLF2 protein, which favors EBV infection. EBV‐infected cells produced virions and exosomes containing the BGLF2 tegument protein. By increasing the expression of the EBV genes and decreasing type I IFN production, exosomal BGLF2 improved the EBV infection. According to these findings, virions and exosomes carrying the viral protein work together to spread EBV [129].

Furthermore, it is important to note that when discussing the role of EVs in EBV biology, we must consider the full spectrum of EVs rather than focusing solely on exosomes. While exosomes (30–150 nm) have been the primary focus of many studies, the size of EBV virions (120–150 nm) necessitates a broader perspective. Microvesicles (50–1000 nm) and apoptotic bodies (800–5000 nm) may also be crucial in EBV‐related intercellular communication and viral pathogenesis. The limitations of current isolation techniques make it challenging to obtain pure populations of specific EV subtypes, further emphasizing the need for a more inclusive approach. While whole virions may be too large for typical exosomal packaging, various EBV components have been detected in EVs, including miRNAs, proteins, and RNA transcripts. These EV‐associated viral factors, rather than complete virions, appear to be the primary mediators of EV‐related effects in EBV infection and associated diseases. Future research should aim to elucidate the distinct roles of different EV subtypes in EBV biology, utilizing advanced isolation techniques and in vivo models to build upon current in vitro findings [130].

### 4.2 HPV and Exosomes

The HPV is a widespread viral infection that comprises a sizable and varied collection of viruses with over 230 well‐known strains [131]. Infected areas of the oropharynx and anogenital regions develop malignant tumors as a result of oncogenic HPVs, particularly high‐risk HPV16 and HPV18. The majority of HPV infections are asymptomatic and resolve on their own [132, 133, 134]. However, some HPV varieties can cause severe illnesses [135]. Given that CC is the fourth most prevalent female cancer overall, the world's health issues confront enormous difficulties. The retinoblastoma and p53 cancer suppressor proteins are only two of the several cell growth regulating pathways that the HPV E6 and E7 oncoproteins target to convert cancer cells [136]. The expression of HPV E6/E7 oncoprotein can probably exert its effect on the microenvironment by controlling the number and composition of miRNAs delivered by exosomes and stimulating the growth of tumor cells [137, 138, 139]. Exosomes from HPV‐positive patients include the oncogenes E6 and E7, according to several studies [140]. Exosome secretion is enhanced in CC, and because they promote angiogenesis, they make metastases more likely [141]. Additionally, the content of exosomes in CC cells is influenced by the HPV‐E6 and E7 virus' oncogenes. Some evidence indicates that exosomes might play an essential function in cancer progression. Research has shown that miR‐21, miR‐146a, and miR‐221 are overexpressed in samples taken by patients with CC in comparison to the samples taken from healthy participants [142]. Furthermore, Wu et al. showed that miR‐221‐3p expression is linked to increased vascular density in tumor microcirculation and is a potent inducer of local angiogenesis [143]. The plasma and cervical samples of CC patients included HPV DNA, a new tumor kinetic marker that exosomes can supply; however, the effect of HPV DNA inside exosomes on the transformation and development of CC remains unknown [144]. In addition to CC, other diseases such as breast cancer (BC) and colorectal cancer are also associated with HPV exosomal DNA. Exosomes can transfer HPV DNA to BC stromal cells, including fibroblasts and epithelial cells, and cause their activation to induce BC cell proliferation and invasion [145]. Wang et al. extracted exosomes from the saliva of HPV‐related oral cancer patients and effectively‐identified HPV16 DNA in salivary exosomes, having 80% consistency with HPV‐16‐positive oral cancer tissues [146]. As a result, HPV‐DNA in salivary exosomes can be used as biomarkers related to HPV‐related CC [147].

### 4.3 HBV and Exosomes

HBV is a significant member of the Hepadnaviridae family of human DNA viruses [148]. Chronic hepatitis B (CHB), cirrhosis, and hepatocellular carcinoma (HCC) are just a few of the devastating liver conditions that can result from the widespread HBV issue [149]. According to Chaudhari et al., HBV uses exosomes to decrease IL‐12 levels and improve infectivity while evading the host's innate immune response [8]. Exosomes from infected hepatocytes include the HBV RNA, viral DNA, core proteAccording to Kouwaki et al., HBV uses exosomes to decrease IL‐12 levels and improve infectivity while evading the host's innate immune response [8]. Exosomes from infectedin, and envelope protein [150]. It is unknown if exosomes produced from hepatocytes infected with HBV mediate the spread of HBV infection [151]. Hepatocytes infected with HBV could reclaim their antiviral status by transferring IFN‐α‐induced antiviral responses from liver nonparenchymal cells to HBV‐infected hepatocytes through exosomes [152]. The HBV X protein (HBx), which is implicated in the replication of the virus and the development of HCC, is supported by an increasing body of data [153]. According to Kapoor et al., HBx alters the exosome's synthesis and uses its inherent intracellular communication capability to transmit the viral load, promoting the pathogenicity and propagation of the virus [154]. The key result was that exosomes were involved in transporting HBV viral particles into the NK cells, accompanied by NK‐cell malfunction caused by upregulation of the inhibitory receptor NKG2A and downregulation of the activating NKp44 receptors. The immunological response is reduced by exosomes associated with HBV [151]. Using exosomes, HBV components can cause immune cell malfunction [155, 156]. According to some research, the ceramide‐dependent mechanism can deliver HBV DNA into exosomes [157]. As a result, the budding and release of exosomes from cells appear similar to that of HBV. Exosomes are believed to contain HBV components such as HBV DNA, HBV RNA, proteins, and even HBV‐miR‐3 [101]. The exosome‐mediated HBV viral component transfer in NK cells alters the cytolytic activity, cytokine production, and cell proliferation by retinoic acid‐inducible gene I (RIG‐I) expression and inactivating the NF‐κB and p38 pathways, which leads to the suppression of the innate immune responses of the liver [151, 154, 155, 158, 159]. Another miRNA that HBV encodes is called HBV‐miR‐3; exosomes and HBV virions in HBV‐infected individuals can release HBV‐miR‐3, which prevents HBV protein and HBV replication [160]. In addition, HBV enhances the production of immunosuppressive miRs, including miR‐21 and miR‐29a in exosomes, which is responsible for a decrease in IL‐12 expression in macrophages, which is necessary for activating NK cells [161]. Exosomes released by HBV‐infected liver cells can reduce apoptosis of tumor cells by increasing exosomal miR‐21 and contributing to chemoresistance and progression of HCC. HBV‐mediated exosomes can participate in chemoresistance in cancer by upregulating a critical protein in chaperone‐mediated autophagy (CMA) called Lamp2a [162]. According to Yang et al., isolated exosomes from CHB patients had a viral component and were infectious to immature hepatocytes [151]. In individuals who have recovered from HBV infection, the exosomes may serve as a reservoir for HBV DNA that can re‐infect the host by suppressing immunity [163]. These investigations corroborate the Trojan exosome concept, which postulates that the virus uses exosomes to speed up its multiplication and transmission while evading the host's innate defensive response [164].

### 4.4 HCV and Exosomes

Infection with the HCV is a leading global cause of chronic liver disease and the accompanying morbidity and death [165, 166]. The majority of the time, asymptomatic chronic HCV infection might result in significant side effects, including cirrhosis or HCC, with approximately 20% of people acquiring HCC [167]. According to several studies, HCV can use exosomes to spread its viral RNA, weaken DCs, and infect naive hepatocytes [24, 150]. Exosomes have also been found to have a role in preventing the innate immune response by neutralizing anti‐HCV antibodies [168]. A recent study found that hepatic exosome‐mediated HCV transmission led to a productive infection [169]. Exosomes of HCV‐infected cells included complete HCV RNA, E2, and HCV core proteins, all of which promote the spread of the virus to new cells and support the capacity of these exosomes to disseminate the virus [23, 24, 150, 170]. According to research, the RAB5A gene may control the proviral content of exosomes, hence controlling the spread of HCC. The capacity of HCC cells to proliferate and migrate can be impacted by RAB5A suppression [171]. The research of Ramakrishnaiah showed that hepatocytes may transmit HCV through exosomes [24]. According to additional research, infections with HCV exosomes demonstrated more HCV transmission to hepatocytes than infections with the same multiplicity of infection (MOI) of free HCV particles [28]. Exosomal HCV RNA is believed to be transported as a complex made up of the proteins Ago2, HSP90, and miR‐122, which has been demonstrated to enhance HCV RNA stability and viral replication [28, 169]. HCV‐related exosomes are implicated in the innate immune response and immunological evasion in addition to the reaction to exosomes harboring HCV RNA [172, 173]. Activation of HSCs has been shown to lead to fibrosis and, thus, the development of end‐stage liver cancer. Due to the lack of miR‐122, cyclophilin A, and ApoE of HCV in HSCs, which are important in the replication and assembly of this virus, these viruses lack the ability to reproduce and produce viral particles [174, 175]. Finally, they cannot activate and induce fibrosis of HSCs [176, 177], so the cause of HSC fibrosis in HCV‐induced liver disorders is not fully understood. However, exosomes appear to be involved in this function. miR‐19a is carried by exosomes produced from HCV‐infected hepatocytes, and these exosomes are also involved in SOCS3 targeting. By doing this, they activate the STAT3‐TGF signaling pathway by HSCs and finally induce fibrosis of these cells [176]. According to research, exosomes from infected cells have been shown to include viral proteins, including Tax and gp46, and cytokines like IL‐10 and IL‐6, Env, HTLV‐1 basic zipper (HBZ), and Tax mRNA transcripts [178].

### 4.5 HTLV‐1 and Exosomes

Adult T‐cell leukemia/lymphoma (ATL) and HTLV‐1‐associated myelopathy/tropical spastic paraparesis (HAM/TSP) are caused by HTLV‐1, which infects persons of all ages worldwide without discrimination. Additionally, HTLV‐1 causes infective dermatitis and HTLV‐1‐associated uveitis [25, 179, 180]. The HBZ protein and two HTLV‐1 regulatory proteins, Tax and Rex, are implicated in the formation of ATLL [181, 182]. Exosomes carrying viral mRNA and miRNAs like tax and hbz are released by HTLV‐1‐infected cells, and these exosomes are responsible for the development of the infection and the inhibition of autophagy in healthy cells [25]. Besides, the exosomal secretory pathway used by HTLV‐1 for infection and dissemination has now been demonstrated. Infected cells express Tax, a trans‐activating protein, within exosomes, according to a study by Alefantis et al. [183]. This protein is transported into infected cells' exosomes by ESCRT‐dependent machinery and is implicated in immunological dysregulation [10]. Cytotoxic T cells and uninfected PBMCs in vitro may live longer when exosomes carrying Tax protein are present. This effect is IL‐2 dependent [10]. Tax protein found in exosomes from HTLV‐1‐infected cells also makes recipient cells susceptible to T lymphocyte lysis [184]. HTLV‐1‐associated neurologic illness (HAM/TSP) patient PBMCs and CSF samples can be used to purify exosomes related to Tax. According to one study, when cocultured with uninfected PBMCs, exosomes from PBMCs of HAM/TSP reduce the CD4 + CD25+ T cells [25, 184]. This information lends credence to the idea that exosomes expressing HTLV‐1 antigen can stimulate inflammatory reactions in HAM/TSP by inhibiting regulatory T cells (Tregs) functionality [185]. The precise function of exosomes in HTLV‐1‐associated illness requires more investigation.

### 4.6 KSHV and Exosomes

Latent and lytic infection stages are separate for the oncogenic herpesvirus known as KSHV in the host. Infected cells keep the viral genome as an extrachromosomal episome during latency [186, 187]. IFI16 acts as a nuclear pathogen sensor during KSHV infection, activating the inflammasome and promoting several inflammatory cytokines (including IL‐1 and IL‐18) to control immunological responses [188]. IFI16 and cleaved IL‐1β are both secreted into exosomes produced by KSHV‐infected cells; therefore, it is most probable that the secretion of these exosomes is a KSHV‐mediated method to undermine IL‐1β functions and weaken immune response [189]. By creating a supportive tumor microenvironment and transforming both infected and noninfected adjacent cells, KSHV‐associated malignancies survive. By significantly modifying the protein and miRNA composition of exosomes from infected cells, KSHV can regulate its microenvironment to promote viral infection and carcinogenesis [190]. According to a prior study, the production of viral genes during viral infections alters the host's miRNA profiles. Additionally, it is known that KSHV generates viral miRNAs that influence host gene expression, aid carcinogenesis, and support viral persistence [191]. KSHV encodes 12 pre‐miRNAs, while 25 mature miRNAs are produced due to subsequent cleavage [192]. These miRNAs regulate EGLN2 and HSPA9 in a cluster to stimulate aerobic glycolysis, such as miR‐K12‐1, 3, 8, and 11, which frequently target EGLN2 and miR‐K12‐1, 3, 4, 6, 8, and 9 which frequently target HSPA9. They also affect the metabolic processes of nearby noninfected cells by exosomal transfer of cellular factors and miRNAs encoded by KSHV [193, 194]. In research, the exosomes of KS patient plasma, pleural effusion, and KS mice models were examined for the presence of circulating cellular and viral miRNAs. They discovered that all of the viral miRNAs are released by KSHV‐infected cells in the form of EVs and that both patient and cultured EVs from KSHV+ lymphomas contain significant concentrations of these viral miRNAs [195]. They suggest that viral miRNAs have effects outside the infected host cell, as KSHV miRNAs were observed in systemically circulating exosomes. It is suggested that these can be introduced as important indicators in KSHV‐related cancers. Using nucleotide patterns called EXOmotifs that promote the loading of miRNAs into exosomes, the classification of specific viral miRNAs is performed by next‐generation sequencing. Then, their accumulation is performed in the exosome of infected cells. A growing body of data suggests that several KSHV miRNAs, including miR‐K12‐4‐3p, miR‐K12‐2‐5p, miR‐K12‐4‐5p, miR‐K12‐6‐5p, miR‐K12‐8‐3p, miR‐K12‐10a‐3p, miR‐K12‐11‐3p, and miR‐K12‐12, are preferentially encapsulated into exosomes released from KSHV‐infected lymphatic [194, 196]. The precise roles of these KSHV miRNAs are currently unclear, and more research is needed. Some key information about oncoviruses and their use of exosomes is summarized in Table 2.

**Table 2:** Some key information about oncoviruses and their use of exosomes.

Oncovirus	Key exosomal cargo	Effects via exosomes	References
EBV	LMP1, LMP2, miRNAs, EBERs	Tumor growth promotion, immune system modulation	[44, 48, 49]
HPV	E6, E7, miRNAs	Tumor cell proliferation, angiogenesis	[77, 78, 82]
HBV	HBV DNA, RNA, proteins	Immune response inhibition, infection spread	[90, 92, 93]
HCV	HCV RNA, proteins	Virus transmission, immune evasion	[107, 109]
HTLV‐1	Tax protein, mRNAs, miRNAs	Infection facilitation, inflammatory reactions	[77, 124]
KSHV	IFI16, IL‐1β, miRNAs	Immune response undermining, tumor microenvironment modification	[130, 132]

## 5 Conclusion

Since the exosome was discovered about 40 years ago, many researchers have become interested in it. Initially, exosomes were suggested as a way to dispose of waste materials in cells. However, later, with several studies, different functions of exosomes were identified. Exosomes are crucial in cancer development, and oncogenic viruses exploit them to accelerate viral pathogenesis. Although viruses are believed to be primary biological organisms, they can play a fiendishly intricate role in cancer development. These examples highlight the various exosomal communication functions crucial for immune system function and cellular microenvironment preservation. The examples in this review also demonstrate how exosomes can boost viral dissemination and the types of cells that can get infected. Exosomes, in general, constitute an intriguing new direction to investigate in viral pathogenesis. Understanding the interaction between exosomes and the tumor microenvironment is crucial for comprehending the incidence, growth, and spread of tumors. It is also crucial for tumor diagnosis, therapy, and prognosis. Exosomes offer much potential as a biomarker for treating, prognosing, and diagnosing several illnesses. Extensive study is required to understand the synthesis of exosomes and distinguish between exosomes that promote the transmission of pathogens and exosomes that activate the host's innate defenses.

## Author Contributions

**Fatemeh Ebrahimi:** writing–review and editing. **Ali Modaresi Movahedi:** writing–review and editing. **Mohammad Sabbaghian:** writing–review and editing. **Vahdat Poortahmasebi:** writing–review and editing, investigation, conceptualization. All authors have read and approved the final manuscript.

## Consent

The authors have nothing to report.

## Conflicts of Interest

The authors declare no conflicts of interest.

## Transparency Statement

The lead author Vahdat Poortahmasebi affirms that this manuscript is an honest, accurate, and transparent account of the study being reported; that no important aspects of the study have been omitted; and that any discrepancies from the study as planned (and, if relevant, registered) have been explained.

## Data Availability

No data was used for the research described in the article.

## References

[1] S. Gurung , D. Perocheau , L. Touramanidou , and J. Baruteau , “The Exosome Journey: From Biogenesis to Uptake and Intracellular Signalling,” Cell Communication and Signaling 19, no. 1 (2021): 47. 33892745 10.1186/s12964-021-00730-1 PMC8063428

[2] L. Doyle and M. Wang , “Overview of Extracellular Vesicles, Their Origin, Composition, Purpose, and Methods for Exosome Isolation and Analysis,” Cells 8, no. 7 (2019): 727. 31311206 10.3390/cells8070727 PMC6678302

[3] C. Dogrammatzis , H. Waisner , and M. Kalamvoki , “Cloaked Viruses and Viral Factors in Cutting Edge Exosome‐Based Therapies,” Frontiers in Cell and Developmental Biology 8 (2020): 376. 32528954 10.3389/fcell.2020.00376 PMC7264115

[4] R. M. Johnstone , M. Adam , J. R. Hammond , L. Orr , and C. Turbide , “Vesicle Formation during Reticulocyte Maturation. Association of Plasma Membrane Activities With Released Vesicles (Exosomes),” Journal of Biological Chemistry 262, no. 19 (1987): 9412–9420. 3597417

[5] C. Théry , L. Zitvogel , and S. Amigorena , “Exosomes: Composition, Biogenesis and Function,” Nature Reviews Immunology 2, no. 8 (2002): 569–579. 10.1038/nri855 12154376

[6] N. Kosaka , “Decoding the Secret of Cancer by Means of Extracellular Vesicles,” Journal of Clinical Medicine 5, no. 2 (2016): 22. 26861408 10.3390/jcm5020022 PMC4773778

[7] K. Takahashi , I. K. Yan , J. Wood , H. Haga , and T. Patel , “Involvement of Extracellular Vesicle Long Noncoding RNA (linc‐VLDLR) in Tumor Cell Responses to Chemotherapy,” Molecular Cancer Research 12, no. 10 (2014): 1377–1387. 24874432 10.1158/1541-7786.MCR-13-0636 PMC4201956

[8] P. Chaudhari , V. Ghate , M. Nampoothiri , and S. Lewis , “Multifunctional Role of Exosomes in Viral Diseases: From Transmission to Diagnosis and Therapy,” Cellular Signalling 94 (2022): 110325. 35367363 10.1016/j.cellsig.2022.110325 PMC8968181

[9] U. Mui , C. Haley , and S. Tyring , “Viral Oncology: Molecular Biology and Pathogenesis,” Journal of Clinical Medicine 6, no. 12 (2017): 111. 29186062 10.3390/jcm6120111 PMC5742800

[10] E. Jaworski , A. Narayanan , R. Van Duyne , et al., “Human T‐Lymphotropic Virus Type 1‐Infected Cells Secrete Exosomes That Contain Tax Protein,” Journal of Biological Chemistry 289, no. 32 (2014): 22284–22305. 24939845 10.1074/jbc.M114.549659 PMC4139239

[11] D. G. Meckes , “Exosomal Communication Goes Viral,” Journal of virology 89, no. 10 (2015): 5200–5203. 25740980 10.1128/JVI.02470-14 PMC4442506

[12] A. Longatti , “The Dual Role of Exosomes in Hepatitis A and C Virus Transmission and Viral Immune Activation,” Viruses 7, no. 12 (2015): 6707–6715. 26694453 10.3390/v7122967 PMC4690890

[13] H. Chahar , X. Bao , and A. Casola , “Exosomes and Their Role in the Life Cycle and Pathogenesis of RNA Viruses,” Viruses 7, no. 6 (2015): 3204–3225. 26102580 10.3390/v7062770 PMC4488737

[14] S.‐Y. Teow , A. C. Nordin , S. A. Ali , and A. S.‐B. Khoo , “Exosomes in Human Immunodeficiency Virus Type I Pathogenesis: Threat or Opportunity?” Advances in Virology 2016 (2016): 1–8. 10.1155/2016/9852494 PMC4766318 26981123

[15] M. Madison and C. Okeoma , “Exosomes: Implications in HIV‐1 Pathogenesis,” Viruses 7, no. 7 (2015): 4093–4118. 26205405 10.3390/v7072810 PMC4517139

[16] S. Y. Teow , K. Liew , A. S. B. Khoo , and S. C. Peh , “Pathogenic Role of Exosomes in Epstein‐Barr Virus (EBV)‐Associated Cancers,” International Journal of Biological Sciences 13, no. 10 (2017): 1276–1286. 29104494 10.7150/ijbs.19531 PMC5666526

[17] D. Iwakiri , “Epstein‐Barr Virus‐Encoded RNAs: Key Molecules in Viral Pathogenesis,” Cancers 6, no. 3 (2014): 1615–1630. 25101570 10.3390/cancers6031615 PMC4190559

[18] A. Canitano , G. Venturi , M. Borghi , M. G. Ammendolia , and S. Fais , “Exosomes Released in Vitro From Epstein–Barr Virus (EBV)‐Infected Cells Contain EBV‐Encoded Latent Phase Mrnas,” Cancer Letters 337, no. 2 (2013): 193–199. 23684926 10.1016/j.canlet.2013.05.012

[19] J. Shen , C. K. Huang , H. Yu , et al., “The Role of Exosomes in Hepatitis, Liver Cirrhosis and Hepatocellular Carcinoma,” Journal of Cellular and Molecular Medicine 21, no. 5 (2017): 986–992. 28224705 10.1111/jcmm.12950 PMC5387156

[20] M. Aga , G. L. Bentz , S. Raffa , et al., “Exosomal HIF1α Supports Invasive Potential of Nasopharyngeal Carcinoma‐Associated LMP1‐Positive Exosomes,” Oncogene 33, no. 37 (2014): 4613–4622. 24662828 10.1038/onc.2014.66 PMC4162459

[21] C. Keryer‐Bibens , C. Pioche‐Durieu , C. Villemant , et al., “Exosomes Released by EBV‐Infected Nasopharyngeal Carcinoma Cells Convey the Viral Latent Membrane Protein 1 and the Immunomodulatory Protein Galectin 9,” BMC Cancer 6, no. 1 (2006): 283. 17156439 10.1186/1471-2407-6-283 PMC1779799

[22] J. Liu , H. Sun , X. Wang , et al., “Increased Exosomal MicroRNA‐21 and MicroRNA‐146a Levels in the Cervicovaginal Lavage Specimens of Patients With Cervical Cancer,” International Journal of Molecular Sciences 15, no. 1 (2014): 758–773. 24406730 10.3390/ijms15010758 PMC3907836

[23] F. Masciopinto , C. Giovani , S. Campagnoli , et al., “Association of Hepatitis C Virus Envelope Proteins With Exosomes,” European Journal of Immunology 34, no. 10 (2004): 2834–2842. 15368299 10.1002/eji.200424887

[24] V. Ramakrishnaiah , C. Thumann , I. Fofana , et al., “Exosome‐Mediated Transmission of Hepatitis C Virus Between Human Hepatoma Huh7.5 Cells,” Proceedings of the National Academy of Sciences of the United States of America 110, no. 32 (2013): 13109–13113. 23878230 10.1073/pnas.1221899110 PMC3740869

[25] D. O. Pinto , C. DeMarino , M. L. Pleet , et al., “HTLV‐1 Extracellular Vesicles Promote Cell‐to‐Cell Contact,” Frontiers in Microbiology 10 (2019): 2147. 31620104 10.3389/fmicb.2019.02147 PMC6759572

[26] D. G. Meckes, Jr. and N. Raab‐Traub , “Microvesicles and Viral Infection,” Journal of Virology 85, no. 24 (2011): 12844–12854. 21976651 10.1128/JVI.05853-11 PMC3233125

[27] J. S. Schorey , Y. Cheng , P. P. Singh , and V. L. Smith , “Exosomes and Other Extracellular Vesicles in Host–Pathogen Interactions,” EMBO Reports 16, no. 1 (2015): 24–43. 25488940 10.15252/embr.201439363 PMC4304727

[28] T. N. Bukong , F. Momen‐Heravi , K. Kodys , S. Bala , and G. Szabo , “Exosomes From Hepatitis C Infected Patients Transmit HCV Infection and Contain Replication Competent Viral RNA in Complex With Ago2‐miR122‐HSP90,” PLoS Pathogens 10, no. 10 (2014): e1004424. 25275643 10.1371/journal.ppat.1004424 PMC4183590

[29] Y. Lee , S. El Andaloussi , and M. J. A. Wood , “Exosomes and Microvesicles: Extracellular Vesicles for Genetic Information Transfer and Gene Therapy,” Human Molecular Genetics 21, no. R1 (2012): R125–R134. 22872698 10.1093/hmg/dds317

[30] S. Principe , A. B. Y. Hui , J. Bruce , A. Sinha , F. F. Liu , and T. Kislinger , “Tumor‐Derived Exosomes and Microvesicles in Head and Neck Cancer: Implications for Tumor Biology and Biomarker Discovery,” Proteomics 13, no. 10–11 (2013): 1608–1623. 23505015 10.1002/pmic.201200533

[31] C. Pan , I. Stevic , V. Müller , et al., “Exosomal MicroRNAs as Tumor Markers in Epithelial Ovarian Cancer,” Molecular Oncology 12, no. 11 (2018): 1935–1948. 30107086 10.1002/1878-0261.12371 PMC6210043

[32] R. Kalluri and V. S. LeBleu , “The Biology, Function, and Biomedical Applications of Exosomes,” Science 367, no. 6478 (2020): eaau6977. 32029601 10.1126/science.aau6977 PMC7717626

[33] S. V. Krylova and D. Feng , “The Machinery of Exosomes: Biogenesis, Release, and Uptake,” International Journal of Molecular Sciences 24, no. 2 (2023): 1337. 36674857 10.3390/ijms24021337 PMC9865891

[34] T. Juan and M. Fürthauer , “Biogenesis and Function of ESCRT‐Dependent Extracellular Vesicles,” Seminars in Cell & Developmental Biology 74 (2018): 66–77. 28807885 10.1016/j.semcdb.2017.08.022

[35] X. Tian , H. Shen , Z. Li , T. Wang , and S. Wang , “Tumor‐Derived Exosomes, Myeloid‐Derived Suppressor Cells, and Tumor Microenvironment,” Journal of Hematology & Oncology 12, no. 1 (2019): 84. 31438991 10.1186/s13045-019-0772-z PMC6704713

[36] B. H. Sung , A. von Lersner , J. Guerrero , et al., “A Live Cell Reporter of Exosome Secretion and Uptake Reveals Pathfinding Behavior of Migrating Cells,” Nature Communications 11, no. 1 (2020): 2092. 10.1038/s41467-020-15747-2 PMC7190671 32350252

[37] Y. Zhang , Y. Liu , H. Liu , and W. H. Tang , “Exosomes: Biogenesis, Biologic Function and Clinical Potential,” Cell & Bioscience 9, no. 1 (2019): 19. 30815248 10.1186/s13578-019-0282-2 PMC6377728

[38] M. F. Baietti , Z. Zhang , E. Mortier , et al., “Syndecan‐Syntenin‐Alix Regulates the Biogenesis of Exosomes,” Nature Cell Biology 14, no. 7 (2012): 677–685. 22660413 10.1038/ncb2502

[39] J. Votteler and W. I. Sundquist , “Virus Budding and the ESCRT Pathway,” Cell Host & Microbe 14, no. 3 (2013): 232–241. 24034610 10.1016/j.chom.2013.08.012 PMC3819203

[40] S. N. Hurwitz , D. Nkosi , M. M. Conlon , et al., “CD63 Regulates Epstein‐Barr Virus LMP1 Exosomal Packaging, Enhancement of Vesicle Production, and Noncanonical NF‐κB Signaling,” Journal of Virology 91, no. 5 (2017): e02251‐16. 27974566 10.1128/JVI.02251-16 PMC5309960

[41] V. Acevedo‐Sánchez , R. M. Rodríguez‐Hernández , S. R. Aguilar‐Ruíz , H. Torres‐Aguilar , and M. A. Romero‐Tlalolini , “Extracellular Vesicles in Cervical Cancer and HPV Infection,” Membranes 11, no. 6 (2021): 453. 34202942 10.3390/membranes11060453 PMC8235012

[42] B. J. Crenshaw , L. Gu , B. Sims , and Q. L. Matthews , “Exosome Biogenesis and Biological Function in Response to Viral Infections,” Open Virology Journal 12 (2018): 134–148. 30416610 10.2174/1874357901812010134 PMC6187740

[43] M. R. Anderson , F. Kashanchi , and S. Jacobson , “Exosomes in Viral Disease,” Neurotherapeutics 13, no. 3 (2016): 535–546. 27324390 10.1007/s13311-016-0450-6 PMC4965413

[44] N. Izquierdo‐Useros , M. Naranjo‐Gómez , I. Erkizia , et al., “HIV and Mature Dendritic Cells: Trojan Exosomes Riding the Trojan Horse?” PLoS Pathogens 6, no. 3 (2010): e1000740. 20360840 10.1371/journal.ppat.1000740 PMC2845607

[45] N. Mardi , S. Haiaty , R. Rahbarghazi , et al., “Exosomal Transmission of Viruses, a Two‐Edged Biological Sword,” Cell Communication and Signaling 21, no. 1 (2023): 19. 36691072 10.1186/s12964-022-01037-5 PMC9868521

[46] A. P. M. Cloherty , A. D. Olmstead , C. M. S. Ribeiro , and F. Jean , “Hijacking of Lipid Droplets by Hepatitis C, Dengue and Zika Viruses—From Viral Protein Moonlighting to Extracellular Release,” International Journal of Molecular Sciences 21, no. 21 (2020): 7901. 33114346 10.3390/ijms21217901 PMC7662613

[47] A. M. Nour and Y. Modis , “Endosomal Vesicles as Vehicles for Viral Genomes,” Trends in Cell Biology 24, no. 8 (2014): 449–454. 24746011 10.1016/j.tcb.2014.03.006 PMC4112135

[48] Y. Yin , Y. Zhao , Q. Chen , Y. Chen , and L. Mao , “Dual Roles and Potential Applications of Exosomes in HCV Infections,” Frontiers in Microbiology 13 (2022): 1044832. 36578571 10.3389/fmicb.2022.1044832 PMC9791051

[49] B. Meng and A. M. L. Lever , “The Interplay Between ESCRT and Viral Factors in the Enveloped Virus Life Cycle,” Viruses 13, no. 2 (2021): 324. 33672541 10.3390/v13020324 PMC7923801

[50] J. Inoue , M. Ninomiya , T. Umetsu , et al., “Small Interfering Rna Screening for the Small GTPase Rab Proteins Identifies Rab5B as a Major Regulator of Hepatitis B Virus Production,” Journal of Virology 93, no. 15 (2019): e00621‐19, 10.1128/jvi.00621-19. 31118260 PMC6639270

[51] L. Zeyen and R. Prange , “Host Cell Rab GTPases in Hepatitis B Virus Infection,” Frontiers in Cell and Developmental Biology 6 (2018): 154. 30510928 10.3389/fcell.2018.00154 PMC6252318

[52] P. P. Gerber , M. Cabrini , C. Jancic , et al., “Rab27a Controls HIV‐1 Assembly by Regulating Plasma Membrane Levels of Phosphatidylinositol 4, 5‐Bisphosphate,” Journal of Cell Biology 209, no. 3 (2015): 435–452. 25940347 10.1083/jcb.201409082 PMC4427790

[53] K. L. McKnight and S. M. Lemon , “Hepatitis A Virus Genome Organization and Replication Strategy,” Cold Spring Harbor Perspectives in Medicine 8, no. 12 (2018): a033480. 29610147 10.1101/cshperspect.a033480 PMC6280712

[54] S. Nagashima , M. Takahashi , N. Jirintai , et al., “A PSAP Motif in the ORF3 Protein of Hepatitis E Virus Is Necessary for Virion Release From Infected Cells,” Journal of General Virology 92, no. 2 (2011): 269–278. 21068219 10.1099/vir.0.025791-0

[55] S. Nagashima , S. Jirintai , M. Takahashi , et al., “Hepatitis E Virus Egress Depends on the Exosomal Pathway, With Secretory Exosomes Derived From Multivesicular Bodies,” Journal of General Virology 95, no. 10 (2014): 2166–2175. 24970738 10.1099/vir.0.066910-0

[56] C. C. P. Celma and P. Roy , “A Viral Nonstructural Protein Regulates Bluetongue Virus Trafficking and Release,” Journal of virology 83, no. 13 (2009): 6806–6816. 19369335 10.1128/JVI.00263-09 PMC2698550

[57] B. Bhattacharya , C. Celma , and P. Roy , “Influence of Cellular Trafficking Pathway on Bluetongue Virus Infection in Ovine Cells,” Viruses 7, no. 5 (2015): 2378–2403. 25984713 10.3390/v7052378 PMC4452911

[58] C. Moulin , M. J. F. Crupi , C. S. Ilkow , J. C. Bell , and S. Boulton , “Extracellular Vesicles and Viruses: Two Intertwined Entities,” International Journal of Molecular Sciences 24, no. 2 (2023): 1036. 36674550 10.3390/ijms24021036 PMC9861478

[59] J. M. Reyes‐Ruiz , J. F. Osuna‐Ramos , L. A. De Jesús‐González , et al., “Isolation and Characterization of Exosomes Released From Mosquito Cells Infected With Dengue Virus,” Virus research 266 (2019): 1–14. 30930201 10.1016/j.virusres.2019.03.015

[60] E. T. Chivero , N. Bhattarai , R. T. Rydze , M. A. Winters , M. Holodniy , and J. T. Stapleton , “Human Pegivirus RNA Is Found in Multiple Blood Mononuclear Cells In Vivo and Serum‐Derived Viral RNA‐Containing Particles Are Infectious In Vitro,” Journal of General Virology 95, no. 6 (2014): 1307–1319. 24668525 10.1099/vir.0.063016-0 PMC4027039

[61] H. Higuchi , N. Yamakawa , K.‐I. Imadome , et al., “Role of Exosomes as a Proinflammatory Mediator in the Development of EBV‐Associated Lymphoma,” Blood 131, no. 23 (2018): 2552–2567. 29685921 10.1182/blood-2017-07-794529

[62] M. I. Costafreda , A. Abbasi , H. Lu , and G. Kaplan , “Exosome Mimicry by a HAVCR1–NPC1 Pathway of Endosomal Fusion Mediates Hepatitis A Virus Infection,” Nature microbiology 5, no. 9 (2020): 1096–1106. 10.1038/s41564-020-0740-y PMC7483988 32541946

[63] I. Kadiu , P. Narayanasamy , P. K. Dash , W. Zhang , and H. E. Gendelman , “Biochemical and Biologic Characterization of Exosomes and Microvesicles as Facilitators of HIV‐1 Infection in Macrophages,” Journal of Immunology 189, no. 2 (2012): 744–754. 10.4049/jimmunol.1102244 PMC3786185 22711894

[64] P. P. Martínez‐Rojas , E. Quiroz‐García , V. Monroy‐Martínez , L. T. Agredano‐Moreno , L. F. Jiménez‐García , and B. H. Ruiz‐Ordaz , “Participation of Extracellular Vesicles From Zika‐Virus‐Infected Mosquito Cells in the Modification of Naïve Cells' Behavior by Mediating Cell‐to‐Cell Transmission of Viral Elements,” Cells 9, no. 1 (2020): 123. 31947958 10.3390/cells9010123 PMC7016930

[65] Y.‐H. Chen , W. Du , M. C. Hagemeijer , et al., “Phosphatidylserine Vesicles Enable Efficient En Bloc Transmission of Enteroviruses,” Cell 160, no. 4 (2015): 619–630. 25679758 10.1016/j.cell.2015.01.032 PMC6704014

[66] S. R. Baglio , M. A. J. van Eijndhoven , D. Koppers‐Lalic , et al., “Sensing of Latent EBV Infection through Exosomal Transfer of 5′ pppRNA,” Proceedings of the National Academy of Sciences of the United States of America 113, no. 5 (2016): E587–E596. 26768848 10.1073/pnas.1518130113 PMC4747727

[67] Y. Fu , L. Zhang , F. Zhang , et al., “Exosome‐Mediated miR‐146a Transfer Suppresses Type I Interferon Response and Facilitates EV71 Infection,” PLoS pathogens 13, no. 9 (2017): e1006611. 28910400 10.1371/journal.ppat.1006611 PMC5614653

[68] M. L. Pleet , C. DeMarino , S. W. Stonier , et al., “Extracellular Vesicles and Ebola Virus: A New Mechanism of Immune Evasion,” Viruses 11, no. 5 (2019): 410. 31052499 10.3390/v11050410 PMC6563240

[69] Y.‐W. Wu , C. Mettling , S.‐R. Wu , et al., “Autophagy‐Associated Dengue Vesicles Promote Viral Transmission Avoiding Antibody Neutralization,” Scientific Reports 6, no. 1 (2016): 32243. 27558165 10.1038/srep32243 PMC4997566

[70] R. D. Wiley and S. Gummuluru , “Immature Dendritic Cell‐Derived Exosomes Can Mediate HIV‐1 Trans Infection,” Proceedings of the National Academy of Sciences of the United States of America 103, no. 3 (2006): 738–743. 16407131 10.1073/pnas.0507995103 PMC1334656

[71] K. Grabowska , M. Wąchalska , M. Graul , M. Rychłowski , K. Bieńkowska‐Szewczyk , and A. D. Lipińska , “Alphaherpesvirus gB Homologs Are Targeted to Extracellular Vesicles, but They Differentially Affect MHC Class II Molecules,” Viruses 12, no. 4 (2020): 429. 32290097 10.3390/v12040429 PMC7232241

[72] A. Caobi , M. Nair , and A. D. Raymond , “Extracellular Vesicles in the Pathogenesis of Viral Infections in Humans,” Viruses 12, no. 10 (2020): 1200. 33096825 10.3390/v12101200 PMC7589806

[73] L. Wang and S. Ning , “New Look of EBV LMP1 Signaling Landscape,” Cancers 13, no. 21 (2021): 5451. 34771613 10.3390/cancers13215451 PMC8582580

[74] J. Flanagan , J. Middeldorp , and T. Sculley , “Localization of the Epstein–Barr Virus Protein LMP 1 to Exosomes,” Journal of General Virology 84, no. 7 (2003): 1871–1879. 12810882 10.1099/vir.0.18944-0

[75] F. S. Y. Yoshikawa , F. M. E. Teixeira , M. N. Sato , and L. M. S. Oliveira , “Delivery of Micrornas by Extracellular Vesicles in Viral Infections: Could the News Be Packaged?” Cells 8, no. 6 (2019): 611. 31216738 10.3390/cells8060611 PMC6627707

[76] T. Liu , X. Zhou , Z. Zhang , et al., “The Role of EBV‐Encoded miRNA in EBV‐Associated Gastric Cancer,” Frontiers in Oncology 13 (2023): 1204030. 37388232 10.3389/fonc.2023.1204030 PMC10301731

[77] D. M. Pegtel , K. Cosmopoulos , D. A. Thorley‐Lawson , et al., “Functional Delivery of Viral miRNAs via Exosomes,” Proceedings of the National Academy of Sciences of the United States of America 107, no. 14 (2010): 6328–6333. 20304794 10.1073/pnas.0914843107 PMC2851954

[78] M. Zieliński , A. Tarasewicz , H. Zielińska , et al., “CD28 Positive, Cytomegalovirus Specific Cytotoxic T Lymphocytes as a Novel Biomarker Associated With Cytomegalovirus Viremia in Kidney Allorecipients,” Journal of Clinical Virology 83 (2016): 17–25. 27526103 10.1016/j.jcv.2016.08.290

[79] E.‐K. Yim and J.‐S. Park , “The Role of HPV E6 and E7 Oncoproteins in HPV‐Associated Cervical Carcinogenesis,” Cancer Research and Treatment 37, no. 6 (2005): 319–324. 19956366 10.4143/crt.2005.37.6.319 PMC2785934

[80] M. V. Chiantore , G. Mangino , M. Iuliano , et al., “Human Papillomavirus E6 and E7 Oncoproteins Affect the Expression of Cancer‐Related MicroRNAs: Additional Evidence in HPV‐Induced Tumorigenesis,” Journal of Cancer Research and Clinical Oncology 142 (2016): 1751–1763. 27300513 10.1007/s00432-016-2189-1 PMC11819219

[81] M. E. Harden and K. Munger , “Human Papillomavirus 16 E6 and E7 Oncoprotein Expression Alters MicroRNA Expression in Extracellular Vesicles,” Virology 508 (2017): 63–69. 28500882 10.1016/j.virol.2017.05.005 PMC5506845

[82] M. H. Van Der Ree , L. Jansen , Z. Kruize , et al., “Plasma Microrna Levels Are Associated With Hepatitis B e Antigen Status and Treatment Response in Chronic Hepatitis B Patients,” Journal of Infectious Diseases 215, no. 9 (2017): 1421–1429. 28368488 10.1093/infdis/jix140

[83] Y. Enomoto , R. Takagi , Y. Naito , et al., “Identification of the Novel 3′ UTR Sequences of Human IL‐21 mRNA as Potential Targets of miRNAs,” Scientific Reports 7, no. 1 (2017): 7780. 28798470 10.1038/s41598-017-07853-x PMC5552845

[84] Z. Klase , P. Kale , R. Winograd , et al., “HIV‐1 TAR Element Is Processed by Dicer to Yield a Viral Micro‐RNA Involved in Chromatin Remodeling of the Viral LTR,” BMC Molecular Biology 8 (2007): 63. 17663774 10.1186/1471-2199-8-63 PMC1955452

[85] A. Narayanan , S. Iordanskiy , R. Das , et al., “Exosomes Derived From HIV‐1‐Infected Cells Contain Trans‐Activation Response Element RNA,” Journal of Biological Chemistry 288, no. 27 (2013): 20014–20033. 23661700 10.1074/jbc.M112.438895 PMC3707700

[86] C. Goh , S. Narayanan , and Y. S. Hahn , “Myeloid‐Derived Suppressor Cells: The Dark Knight or the Joker in Viral Infections?” Immunological Reviews 255, no. 1 (2013): 210–221. 23947357 10.1111/imr.12084 PMC3748397

[87] G. C. Sampey , M. Saifuddin , A. Schwab , et al., “Exosomes From HIV‐1‐infected Cells Stimulate Production of Pro‐Inflammatory Cytokines Through Trans‐Activating Response (TAR) RNA,” Journal of Biological Chemistry 291, no. 3 (2016): 1251–1266. 26553869 10.1074/jbc.M115.662171 PMC4714213

[88] F. Giannessi , A. Aiello , F. Franchi , Z. A. Percario , and E. Affabris , “The Role of Extracellular Vesicles as Allies of HIV, HCV and SARS Viruses,” Viruses 12, no. 5 (2020): 571. 32456011 10.3390/v12050571 PMC7291340

[89] J. H. Kim , C. H. Lee , and S.‐W. Lee , “Exosomal Transmission of MicroRNA From HCV Replicating Cells Stimulates Transdifferentiation in Hepatic Stellate Cells,” Molecular Therapy‐Nucleic Acids 14 (2019): 483–497. 30753992 10.1016/j.omtn.2019.01.006 PMC6369229

[90] M. Panigrahi , M. A. Palmer , and J. A. Wilson , “MicroRNA‐122 Regulation of HCV Infections: Insights From Studies of miR‐122‐Independent Replication,” Pathogens 11, no. 9 (2022): 1005. 36145436 10.3390/pathogens11091005 PMC9504723

[91] L. Urbanelli , S. Buratta , B. Tancini , et al., “The Role of Extracellular Vesicles in Viral Infection and Transmission,” Vaccines 7, no. 3 (2019): 102. 31466253 10.3390/vaccines7030102 PMC6789493

[92] J. Sadri Nahand , F. Bokharaei‐Salim , M. Karimzadeh , et al., “MicroRNAs and Exosomes: Key Players in HIV Pathogenesis,” HIV Medicine 21, no. 4 (2020): 246–278. 31756034 10.1111/hiv.12822 PMC7069804

[93] A. Kumar , S. Kodidela , E. Tadrous , et al., “Extracellular Vesicles in Viral Replication and Pathogenesis and Their Potential Role in Therapeutic Intervention,” Viruses 12, no. 8 (2020): 887. 32823684 10.3390/v12080887 PMC7472073

[94] D. G. Meckes, Jr. , H. P. Gunawardena , R. M. Dekroon , P. R. Heaton , R. H. Edwards , and S. Ozgur , “Modulation of B‐Cell Exosome Proteins by Gamma Herpesvirus Infection,” Proceedings of the National Academy of Sciences of the United States of America 110, no. 31 (2013): E2925–E2933. 23818640 10.1073/pnas.1303906110 PMC3732930

[95] H. Mohamed , T. Gurrola , R. Berman , et al., “Targeting CCR5 as a Component of an HIV‐1 Therapeutic Strategy,” Frontiers in Immunology 12 (2021): 816515. 35126374 10.3389/fimmu.2021.816515 PMC8811197

[96] J. Wang , S. Chen , and J. Bihl , “Exosome‐Mediated Transfer of ACE2 (Angiotensin‐Converting Enzyme 2) From Endothelial Progenitor Cells Promotes Survival and Function of Endothelial Cell,” Oxidative Medicine and Cellular Longevity 2020, no. 1 (2020): 4213541. 32051731 10.1155/2020/4213541 PMC6995312

[97] P. Gangadaran , H. Madhyastha , R. Madhyastha , et al., “The Emerging Role of Exosomes in Innate Immunity, Diagnosis and Therapy,” Frontiers in Immunology 13 (2022): 1085057. 36726968 10.3389/fimmu.2022.1085057 PMC9885214

[98] E. Karamichali , P. Foka , G. Papadopoulou , et al., “Hepatitis Viruses Control Host Immune Responses by Modifying the Exosomal Biogenesis Pathway and Cargo,” International Journal of Molecular Sciences 23, no. 18 (2022): 10862. 36142773 10.3390/ijms231810862 PMC9505460

[99] M. A. Kumar , S. K. Baba , H. Q. Sadida , et al., “Extracellular Vesicles as Tools and Targets in Therapy for Diseases,” Signal Transduction and Targeted Therapy 9, no. 1 (2024): 27. 38311623 10.1038/s41392-024-01735-1 PMC10838959

[100] M. Koch , H.‐J. Mollenkopf , U. Klemm , and T. F. Meyer , “Induction of MicroRNA‐155 Is TLR‐and Type IV Secretion System‐Dependent in Macrophages and Inhibits DNA‐Damage Induced Apoptosis,” Proceedings of the National Academy of Sciences of the United States of America 109, no. 19 (2012): E1153–E1162. 22509021 10.1073/pnas.1116125109 PMC3358876

[101] Y. Peng , Y. Yang , Y. Li , T. Shi , Y. Luan , and C. Yin , “Exosome and Virus Infection,” Frontiers in Immunology 14 (2023): 1154217. 37063897 10.3389/fimmu.2023.1154217 PMC10098074

[102] N. Raab‐Traub and D. P. Dittmer , “Viral Effects on the Content and Function of Extracellular Vesicles,” Nature Reviews Microbiology 15, no. 9 (2017): 559–572. 28649136 10.1038/nrmicro.2017.60 PMC5555775

[103] D. G. Meckes, Jr. , K. H. Y. Shair , A. R. Marquitz , C.‐P. Kung , R. H. Edwards , and N. Raab‐Traub , “Human Tumor Virus Utilizes Exosomes for Intercellular Communication,” Proceedings of the National Academy of Sciences of the United States of America 107, no. 47 (2010): 20370–20375. 21059916 10.1073/pnas.1014194107 PMC2996715

[104] A. Nanbo , E. Kawanishi , R. Yoshida , and H. Yoshiyama , “Exosomes Derived From Epstein‐Barr Virus‐Infected Cells Are Internalized via Caveola‐Dependent Endocytosis and Promote Phenotypic Modulation in Target Cells,” Journal of virology 87, no. 18 (2013): 10334–10347. 23864627 10.1128/JVI.01310-13 PMC3753980

[105] E. Kieff and A. B. Rickinson , “Epstein‐Barr Virus and Its Replication,” in Fields Virology, 5th ed., vol. II, eds. D. M., Knipe , P. M., Howley , D. E., Griffin , R. A., Lamb , M. M., Martin , B. Roizman , and S. E., Straus (Philadelphia: Lippincott Williams & Wilkins, 2007), 2603–2654.

[106] W. Fang , J. Zhang , S. Hong , et al., “EBV‐Driven LMP1 and IFN‐γ Up‐Regulate PD‐L1 in Nasopharyngeal Carcinoma: Implications for Oncotargeted Therapy,” Oncotarget 5, no. 23 (2014): 12189–12202. 25361008 10.18632/oncotarget.2608 PMC4322961

[107] N. Wakisaka , S. Kondo , T. Yoshizaki , S. Murono , M. Furukawa , and J. S. Pagano , “Epstein‐Barr Virus Latent Membrane Protein 1 Induces Synthesis of Hypoxia‐Inducible Factor 1Α,” Molecular and Cellular Biology 24, no. 12 (2004): 5223–5234. 15169887 10.1128/MCB.24.12.5223-5234.2004 PMC419879

[108] B. Kim and K.‐M. Kim , “Role of Exosomes and Their Potential as Biomarkers in Epstein–Barr Virus‐Associated Gastric Cancer,” Cancers 15, no. 2 (2023): 469. 36672418 10.3390/cancers15020469 PMC9856651

[109] G. Raposo and W. Stoorvogel , “Extracellular Vesicles: Exosomes, Microvesicles, and Friends,” Journal of Cell Biology 200, no. 4 (2013): 373–383. 23420871 10.1083/jcb.201211138 PMC3575529

[110] E. Kobayashi , M. Aga , S. Kondo , et al., “C‐Terminal Farnesylation of UCH‐L1 Plays a Role in Transport of Epstein‐Barr Virus Primary Oncoprotein LMP1 to Exosomes,” Msphere 3, no. 1 (2018): e00030‐18. 29435490 10.1128/mSphere.00030-18 PMC5806207

[111] W. Ahmed , S. Tariq , and G. Khan , “Tracking EBV‐Encoded RNAs (EBERs) From the Nucleus to the Excreted Exosomes of B‐Lymphocytes,” Scientific Reports 8, no. 1 (2018): 15438. 30337610 10.1038/s41598-018-33758-4 PMC6193935

[112] L. Zuo , Y. Xie , J. Tang , et al., “Targeting Exosomal EBV‐LMP1 Transfer and miR‐203 Expression via the NF‐κB Pathway: the Therapeutic Role of Aspirin in NPC,” Molecular Therapy‐Nucleic Acids 17 (2019): 175–184. 31265948 10.1016/j.omtn.2019.05.023 PMC6610683

[113] D. M. Pegtel , M. D. B. van de Garde , and J. M. Middeldorp , “Viral Mirnas Exploiting the Endosomal–Exosomal Pathway for Intercellular Cross‐Talk and Immune Evasion,” Biochimica et Biophysica Acta—Gene Regulatory Mechanisms 1809, no. 11 (2011): 715–721. 10.1016/j.bbagrm.2011.08.002 21855666

[114] C. W. Dawson , R. J. Port , and L. S. Young , “The Role of the EBV‐Encoded Latent Membrane Proteins LMP1 and LMP2 in the Pathogenesis of Nasopharyngeal Carcinoma (NPC),” Seminars in Cancer Biology 22, no. 2 (2012): 144–153. 22249143 10.1016/j.semcancer.2012.01.004

[115] S. W. Tsao , C. M. Tsang , K. F. To , and K. W. Lo , “The Role of Epstein–Barr Virus in Epithelial Malignancies,” Journal of Pathology 235 (2015): 323–333. 25251730 10.1002/path.4448 PMC4280676

[116] X. Huang , M. Zhang , and Z. Zhang , “The Role of LMP1 in Epstein‐Barr Virus‐Associated Gastric Cancer,” Current Cancer Drug Targets 24, no. 2 (2024): 127–141. 37183458 10.2174/1568009623666230512153741

[117] D. Mrizak , N. Martin , C. Barjon , et al., “Effect of Nasopharyngeal Carcinoma‐Derived Exosomes on Human Regulatory T Cells,” Journal of the National Cancer Institute 107, no. 1 (2014): 363. 25505237 10.1093/jnci/dju363

[118] J. Klibi , T. Niki , A. Riedel , et al., “Blood Diffusion and Th1‐suppressive Effects of Galectin‐9‐Containing Exosomes Released by Epstein‐Barr Virus‐Infected Nasopharyngeal Carcinoma Cells,” Blood 113, no. 9 (2009): 1957–1966. 19005181 10.1182/blood-2008-02-142596

[119] D. F. Dukers , P. Meij , M. B. Vervoort , et al., “Direct Immunosuppressive Effects of EBV‐Encoded Latent Membrane Protein 1,” Journal of Immunology 165, no. 2 (2000): 663–670. 10.4049/jimmunol.165.2.663 10878338

[120] S. Ceccarelli , V. Visco , S. Raffa , N. Wakisaka , J. S. Pagano , and M. R. Torrisi , “Epstein‐Barr Virus Latent Membrane Protein 1 Promotes Concentration in Multivesicular Bodies of Fibroblast Growth Factor 2 and Its Release Through Exosomes,” International Journal of Cancer 121, no. 7 (2007): 1494–1506. 17546597 10.1002/ijc.22844

[121] S. N. Hurwitz , M. R. Cheerathodi , D. Nkosi , S. B. York , and D. G. Meckes Jr. , “Tetraspanin CD63 Bridges Autophagic and Endosomal Processes to Regulate Exosomal Secretion and Intracellular Signaling of Epstein‐Barr Virus LMP1,” Journal of Virology 92, no. 5 (2018): e01969‐17. 29212935 10.1128/JVI.01969-17 PMC5809724

[122] W. Ahmed , P. S. Philip , S. Tariq , and G. Khan , “Epstein‐Barr Virus‐Encoded Small RNAs (EBERs) Are Present in Fractions Related to Exosomes Released by EBV‐Transformed Cells,” PLoS One 9, no. 6 (2014): e99163. 24896633 10.1371/journal.pone.0099163 PMC4045842

[123] W. Ahmed and G. Khan , “The Labyrinth of Interactions of Epstein‐Barr Virus‐Encoded Small RNAs,” Reviews in Medical Virology 24, no. 1 (2014): 3–14. 24105992 10.1002/rmv.1763

[124] D. Iwakiri and K. Takada , “Role of EBERs in the Pathogenesis of EBV Infection,” Advances in Cancer Research 107 (2010): 119–136. 20399962 10.1016/S0065-230X(10)07004-1

[125] A. Canitano , G. Venturi , M. Borghi , M. G. Ammendolia , and S. Fais , “Exosomes Released in Vitro From Epstein‐Barr Virus (EBV)‐Infected Cells Contain EBV‐Encoded Latent Phase mRNAs,” Cancer letters 337, no. 2 (2013): 193–199. 23684926 10.1016/j.canlet.2013.05.012

[126] F. J. Verweij , M. A. J. van Eijndhoven , J. M. Middeldorp , and D. M. Pegtel , “Analysis of Viral MicroRNA Exchange via Exosomes In Vitro and In Vivo,” Methods in molecular biology 1024 (2013): 53–68. 23719942 10.1007/978-1-62703-453-1_5

[127] R. Rasizadeh , P. S. Aghbash , J. S. Nahand , T. Entezari‐Maleki , and H. B. Baghi , “SARS‐CoV‐2‐Associated Organs Failure and Inflammation: A Focus on the Role of Cellular and Viral MicroRNAs,” Virology Journal 20, no. 1 (2023): 179. 37559103 10.1186/s12985-023-02152-6 PMC10413769

[128] H. Vallhov , C. Gutzeit , S. M. Johansson , et al., “Exosomes Containing Glycoprotein 350 Released by EBV‐Transformed B Cells Selectively Target B Cells Through CD21 and Block EBV Infection in Vitro,” Journal of Immunology 186, no. 1 (2011): 73–82. 10.4049/jimmunol.1001145 21106852

[129] Y. Sato , M. Yaguchi , Y. Okuno , et al., “Epstein–Barr Virus Tegument Protein BGLF2 in Exosomes Released From Virus‐Producing Cells Facilitates De Novo Infection,” Cell Communication and Signaling 20, no. 1 (2022): 95. 35729616 10.1186/s12964-022-00902-7 PMC9210680

[130] M. Zhao , A. Nanbo , L. Sun , and Z. Lin , “Extracellular Vesicles in Epstein‐Barr Virus' Life Cycle and Pathogenesis,” Microorganisms 7, no. 2 (2019): 48. 30754656 10.3390/microorganisms7020048 PMC6406486

[131] E. F. Dunne , E. R. Unger , M. Sternberg , et al., “Prevalence of HPV Infection Among Females in the United States,” JAMA 297, no. 8 (2007): 813–819. 17327523 10.1001/jama.297.8.813

[132] H. zur Hausen , “Papillomaviruses and Cancer: From Basic Studies to Clinical Application,” Nature Reviews Cancer 2, no. 5 (2002): 342–350. 12044010 10.1038/nrc798

[133] P. Pinidis , P. Tsikouras , G. Iatrakis , et al., “Human Papilloma Virus' Life Cycle and Carcinogenesis,” Maedica 11, no. 1 (2016): 48–54. 28465751 PMC5394500

[134] S. M. Mousavi , S. A. Hashemi , S. Bahrani , et al., “Recent Advancements in Polythiophene‐Based Materials and Their Biomedical, Geno Sensor and DNA Detection,” International Journal of Molecular Sciences 22 (2021): 6850. 34202199 10.3390/ijms22136850 PMC8268102

[135] P. Pinidis , P. Tsikouras , G. Iatrakis , et al., “Retraction: Human Papilloma Virus' Life Cycle and Carcinogenesis,” Maedica 13, no. 1 (2018): 85. 35440947 PMC8984816

[136] D. Urban , J. Corry , and D. Rischin , “What Is the Best Treatment for Patients With Human Papillomavirus‐Positive and ‐Negative Oropharyngeal Cancer?” Cancer 120 (2014): 1462–1470. 24578320 10.1002/cncr.28595

[137] A. Honegger , D. Schilling , H. Sültmann , K. Hoppe‐Seyler , and F. Hoppe‐Seyler , “Identification of E6/E7‐Dependent MicroRNAs in HPV‐Positive Cancer Cells,” Methods in Molecular Biology 1699 (2018): 119–134. 29086374 10.1007/978-1-4939-7435-1_10

[138] M. V. Chiantore , G. Mangino , M. Iuliano , et al., “Human Papillomavirus and Carcinogenesis: Novel Mechanisms of Cell Communication Involving Extracellular Vesicles,” Cytokine & Growth Factor Reviews 51 (2020): 92–98. 31973992 10.1016/j.cytogfr.2019.12.009 PMC7108386

[139] J. Wu , J. Yang , J. Ding , X. Guo , X. Q. Zhu , and Y. Zheng , “Exosomes in Virus‐Associated Cancer,” Cancer Letters 438 (2018): 44–51. 30219505 10.1016/j.canlet.2018.09.018

[140] A. Honegger , D. Schilling , S. Bastian , et al., “Dependence of Intracellular and Exosomal MicroRNAs on Viral E6/E7 Oncogene Expression in HPV‐Positive Tumor Cells,” PLoS Pathogens 11, no. 3 (2015): e1004712. 25760330 10.1371/journal.ppat.1004712 PMC4356518

[141] A. Bhat , J. Yadav , K. Thakur , et al., “Exosomes From Cervical Cancer Cells Facilitate Pro‐Angiogenic Endothelial Reconditioning Through Transfer of Hedgehog‐GLI Signaling Components,” Cancer Cell International 21, no. 1 (2021): 319. 34167524 10.1186/s12935-021-02026-3 PMC8223267

[142] S. Esfandyari , H. Elkafas , R. M. Chugh , H.‐s Park , A. Navarro , and A. Al‐Hendy , “Exosomes as Biomarkers for Female Reproductive Diseases Diagnosis and Therapy,” International Journal of Molecular Sciences 22, no. 4 (2021): 2165. 33671587 10.3390/ijms22042165 PMC7926632

[143] X. Wu , C. Zhou , Y.‐M. Zhang , et al., “Cancer‐Derived Exosomal miR‐221‐3p Promotes Angiogenesis by Targeting THBS2 in Cervical Squamous Cell Carcinoma,” Angiogenesis 22 (2019): 397–410. 30993566 10.1007/s10456-019-09665-1

[144] M. Mata‐Rocha , R. M. Rodríguez‐Hernández , P. Chávez‐Olmos , et al., “Presence of HPV DNA in Extracellular Vesicles From Hela Cells and Cervical Samples,” Enfermedades Infecciosas y Microbiología Clínica 38, no. 4 (2020): 159–165. 31395428 10.1016/j.eimc.2019.06.011

[145] S. De Carolis , G. Storci , C. Ceccarelli , et al., “HPV DNA Associates With Breast Cancer Malignancy and It Is Transferred to Breast Cancer Stromal Cells by Extracellular Vesicles,” Frontiers in Oncology 9 (2019): 860. 31608222 10.3389/fonc.2019.00860 PMC6756191

[146] Z. Wang , F. Li , J. Rufo , et al, “Acoustofluidic Salivary Exosome Isolation: A Liquid Biopsy Compatible Approach for Human Papillomavirus‐Associated Oropharyngeal Cancer Detection.” Journal of Molecular Diagnostics (2019). 10.1016/j.jmoldx.2019.08.004 PMC6943372 31843276

[147] C. Ekanayake Weeramange , Z. Liu , G. Hartel , et al., “Salivary High‐Risk Human Papillomavirus (HPV) DNA as a Biomarker for HPV‐Driven Head and Neck Cancers,” Journal of Molecular Diagnostics 23, no. 10 (2021): 1334–1342. 10.1016/j.jmoldx.2021.07.005 PMC9344924 34325059

[148] M. Bandopadhyay and M. Bharadwaj , “Exosomal miRNAs in Hepatitis B Virus Related Liver Disease: A New Hope for Biomarker,” Gut Pathogens 12 (2020): 23. 32346400 10.1186/s13099-020-00353-w PMC7183117

[149] A. Arzumanyan , H. M. G. P. V. Reis , and M. A. Feitelson , “Pathogenic Mechanisms in HBV‐ and HCV‐Associated Hepatocellular Carcinoma,” Nature Reviews Cancer 13, no. 2 (2013): 123–135. 23344543 10.1038/nrc3449

[150] K. Tamai , M. Shiina , N. Tanaka , et al., “Regulation of Hepatitis C Virus Secretion by the Hrs‐Dependent Exosomal Pathway,” Virology 422, no. 2 (2012): 377–385. 22138215 10.1016/j.virol.2011.11.009

[151] Y. Yang , Q. Han , Z. Hou , C. Zhang , Z. Tian , and J. Zhang , “Exosomes Mediate Hepatitis B Virus (HBV) Transmission and NK‐Cell Dysfunction,” Cellular & Molecular Immunology 14, no. 5 (2017): 465–475. 27238466 10.1038/cmi.2016.24 PMC5423088

[152] J. Li , K. Liu , Y. Liu , et al., “Exosomes Mediate the Cell‐To‐Cell Transmission of IFN‐α‐induced Antiviral Activity,” Nature Immunology 14, no. 8 (2013): 793–803. 23832071 10.1038/ni.2647

[153] L. Deng , X. Gan , M. Ito , et al., “Peroxiredoxin 1, a Novel HBx‐Interacting Protein, Interacts With Exosome Component 5 and Negatively Regulates Hepatitis B Virus (HBV) Propagation through Degradation of HBV RNA,” Journal of Virology 93, no. 6 (2019): e02203‐18. 30567989 10.1128/JVI.02203-18 PMC6401444

[154] N. R. Kapoor , R. Chadha , S. Kumar , T. Choedon , V. S. Reddy , and V. Kumar , “The HBx Gene of Hepatitis B Virus Can Influence Hepatic Microenvironment via Exosomes by Transferring Its mRNA and Protein,” Virus Research 240 (2017): 166–174. 28847700 10.1016/j.virusres.2017.08.009

[155] J. Wang , D. Cao , and J. Yang , “Exosomes in Hepatitis B Virus Transmission and Related Immune Response,” Tohoku Journal of Experimental Medicine 252, no. 4 (2020): 309–320. 33268600 10.1620/tjem.252.309

[156] S. Teymouri , M. Pourhajibagher , and A. Bahador , “Exosomes: Friends or Foes in Microbial Infections?,” Infectious Disorders—Drug Targets 24 (2024): 27–44. 10.2174/0118715265264388231128045954 38317472

[157] T. Sanada , Y. Hirata , Y. Naito , et al., “Transmission of HBV DNA Mediated by Ceramide‐Triggered Extracellular Vesicles,” Cellular and Molecular Gastroenterology and Hepatology 3, no. 2 (2017): 272–283. 28275693 10.1016/j.jcmgh.2016.10.003 PMC5331779

[158] T. Y. Li , Y. Yang , G. Zhou , and Z. K. Tu , “Immune Suppression in Chronic Hepatitis B Infection Associated Liver Disease: A Review,” World Journal of Gastroenterology 25, no. 27 (2019): 3527–3537. 31367154 10.3748/wjg.v25.i27.3527 PMC6658392

[159] X. Jia , J. Chen , D. A. Megger , et al., “Label‐Free Proteomic Analysis of Exosomes Derived From Inducible Hepatitis B Virus‐Replicating HepAD38 Cell Line,” supplement Molecular & Cellular Proteomics 16, no. 4 S1 (2017): S144–S160. 28242843 10.1074/mcp.M116.063503 PMC5393393

[160] X. Yang , H. Li , H. Sun , et al., “Hepatitis B Virus‐Encoded MicroRNA Controls Viral Replication,” Journal of Virology 91, no. 10 (2017): e01919‐16. 28148795 10.1128/JVI.01919-16 PMC5411615

[161] T. Kouwaki , Y. Fukushima , T. Daito , et al., “Extracellular Vesicles Including Exosomes Regulate Innate Immune Responses to Hepatitis B Virus Infection,” Frontiers in Immunology 7 (2016): 335. 27630638 10.3389/fimmu.2016.00335 PMC5005343

[162] D. X. Liu , P.‐p Li , J. P. Guo , et al., “Exosomes Derived From HBV‐Associated Liver Cancer Promote Chemoresistance by Upregulating Chaperone‐Mediated Autophagy,” Oncology Letters 17 (2019): 323–331. 30655770 10.3892/ol.2018.9584 PMC6313222

[163] S. Sukriti , J. S. Maras , C. Bihari , et al., “Microvesicles in Hepatic and Peripheral Vein Can Predict Nonresponse to Corticosteroid Therapy in Severe Alcoholic Hepatitis,” Alimentary Pharmacology & Therapeutics 47 (2018): 1151–1161. 29460445 10.1111/apt.14564

[164] S. J. Gould , A. M. Booth , and J. E. Hildreth , “The Trojan Exosome Hypothesis,” Proceedings of the National Academy of Sciences of the United States of America 100, no. 19 (2003): 10592–10597. 12947040 10.1073/pnas.1831413100 PMC196848

[165] T. Vos , R. M. Barber , B. Bell , et al., “Global, Regional, and National Incidence, Prevalence, and Years Lived With Disability for 301 Acute and Chronic Diseases and Injuries in 188 Countries, 1990‐2013: A Systematic Analysis for the Global Burden of Disease Study 2013,” Lancet 386, no. 9995 (2015): 743–800. 26063472 10.1016/S0140-6736(15)60692-4 PMC4561509

[166] G. Indolfi , P. Easterbrook , G. Dusheiko , et al., “Hepatitis C Virus Infection in Children and Adolescents,” Lancet Gastroenterology & Hepatology 4, no. 6 (2019): 477–487. 30982721 10.1016/S2468-1253(19)30046-9

[167] A. Petruzziello , S. Marigliano , G. Loquercio , A. Cozzolino , and C. Cacciapuoti , “Global Epidemiology of Hepatitis C Virus Infection: An Up‐Date of the Distribution and Circulation of Hepatitis C Virus Genotypes,” World Journal of Gastroenterology 22, no. 34 (2016): 7824–7840. 27678366 10.3748/wjg.v22.i34.7824 PMC5016383

[168] J. Wang , Y. Yao , X. Chen , J. Wu , T. Gu , and X. Tang , “Host Derived Exosomes‐Pathogens Interactions: Potential Functions of Exosomes in Pathogen Infection,” Biomedicine & Pharmacotherapy 108 (2018): 1451–1459. 30372847 10.1016/j.biopha.2018.09.174

[169] F. L. Cosset and M. Dreux , “HCV Transmission by Hepatic Exosomes Establishes a Productive Infection,” Journal of Hepatology 60, no. 3 (2014): 674–675. 24512825 10.1016/j.jhep.2013.10.015

[170] Z. Liu , X. Zhang , Q. Yu , and J. J. He , “Exosome‐Associated Hepatitis C Virus in Cell Cultures and Patient Plasma,” Biochemical and Biophysical Research Communications 455, no. 3–4 (2014): 218–222. 25449270 10.1016/j.bbrc.2014.10.146

[171] G. Gorji‐bahri , H. R. Moghimi , and A. Hashemi , “RAB5A Effect on Metastasis of Hepatocellular Carcinoma Cell Line via Altering the Pro‐Invasive Content of Exosomes,” Experimental and Molecular Pathology 120 (2021): 104632. 33831402 10.1016/j.yexmp.2021.104632

[172] M. Dreux , U. Garaigorta , B. Boyd , et al., “Short‐Range Exosomal Transfer of Viral RNA From Infected Cells to Plasmacytoid Dendritic Cells Triggers Innate Immunity,” Cell Host & Microbe 12, no. 4 (2012): 558–570. 23084922 10.1016/j.chom.2012.08.010 PMC3479672

[173] K. Takahashi , S. Asabe , S. Wieland , et al., “Plasmacytoid Dendritic Cells Sense Hepatitis C Virus–Infected Cells, Produce Interferon, and Inhibit Infection,” Proceedings of the National Academy of Sciences of the United States of America 107 (2010): 7431–7436. 20231459 10.1073/pnas.1002301107 PMC2867703

[174] C. L. Jopling , M. Yi , A. M. Lancaster , S. M. Lemon , and P. Sarnow , “Modulation of Hepatitis C Virus RNA Abundance by a Liver‐Specific MicroRNA,” Science 309, no. 5740 (2005): 1577–1581. 16141076 10.1126/science.1113329

[175] G. Long , M.‐S. Hiet , M. P. Windisch , J.‐Y. Lee , V. Lohmann , and R. Bartenschlager , “Mouse Hepatic Cells Support Assembly of Infectious Hepatitis C Virus Particles,” Gastroenterology 141, no. 3 (2011): 1057–1066. 21699799 10.1053/j.gastro.2011.06.010

[176] P. B. Devhare , R. Sasaki , S. Shrivastava , A. M. Di Bisceglie , R. Ray , and R. B. Ray , “Correction for Devhare et al., “Exosome‐Mediated Intercellular Communication between Hepatitis C Virus‐Infected Hepatocytes and Hepatic Stellate Cells”,” Journal of Virology 91, no. 10 (2017): e02225‐16. 28455354 10.1128/JVI.00349-17 PMC5411579

[177] A. Florimond , P. Chouteau , P. Bruscella , et al., “Human Hepatic Stellate Cells Are Not Permissive for Hepatitis C Virus Entry and Replication,” Gut 64 (2014): 957–965. 25063678 10.1136/gutjnl-2013-305634

[178] A. Narayanan , E. Jaworski , R. Van Duyne , et al., “Exosomes Derived From HTLV‐1 Infected Cells Contain the Viral Protein Tax,” Retrovirology 11, no. 1 (2014): O46.

[179] F. Martin , Y. Tagaya , and R. Gallo , “Time to Eradicate HTLV‐1: An Open Letter to WHO,” Lancet 391 (2018): 1893–1894. 29781438 10.1016/S0140-6736(18)30974-7

[180] A. Gessain , J. C. Vernant , L. Maurs , et al., “Antibodies to Human T‐Lymphotropic Virus Type‐I in Patients With Tropical Spastic Paraparesis,” Lancet 326, no. 8452 (1985): 407–410. 10.1016/s0140-6736(85)92734-5 2863442

[181] S. Philip , M. A. Zahoor , H. Zhi , Y.‐K. Ho , and C.‐Z. Giam , “Regulation of Human T‐Lymphotropic Virus Type I Latency and Reactivation by HBZ and Rex,” PLoS Pathogens 10 (2014): e1004040. 24699669 10.1371/journal.ppat.1004040 PMC3974842

[182] A. Alasiri , J. Abboud Guerr , W. W. Hall , and N. Sheehy , “Novel Interactions between the Human T‐Cell Leukemia Virus Type 1 Antisense Protein HBZ and the SWI/SNF Chromatin Remodeling Family: Implications for Viral Life Cycle,” Journal of Virology 93, no. 16 (2019): e00412‐19. 31142665 10.1128/JVI.00412-19 PMC6675893

[183] T. Alefantis , K. Mostoller , P. Jain , E. Harhaj , C. Grant , and B. Wigdahl , “Secretion of the Human T Cell Leukemia Virus Type I Transactivator Protein Tax,” Journal of Biological Chemistry 280 (2005): 17353–17362. 15659397 10.1074/jbc.M409851200

[184] M. R. Anderson , M. L. Pleet , Y. Enose‐Akahata , et al., “Viral Antigens Detectable in CSF Exosomes From Patients With Retrovirus Associated Neurologic Disease: Functional Role of Exosomes,” Clinical and Translational Medicine 7, no. 1 (2018): 24. 30146667 10.1186/s40169-018-0204-7 PMC6110307

[185] I. S. Okoye , S. M. Coomes , V. S. Pelly , et al., “MicroRNA‐Containing T‐Regulatory‐Cell‐Derived Exosomes Suppress Pathogenic T Helper 1 Cells,” Immunity 41, no. 3 (2014): 503. 28903020 10.1016/j.immuni.2014.08.008 PMC5640441

[186] P. K. Sandhu and B. Damania , “The Regulation of KSHV Lytic Reactivation by Viral and Cellular Factors,” Current Opinion in Virology 52 (2022): 39–47. 34872030 10.1016/j.coviro.2021.11.004 PMC8844089

[187] C. Han , D. Zhang , C. Gui , et al., “KSHV RTA Antagonizes SMC5/6 Complex‐Induced Viral Chromatin Compaction by Hijacking the Ubiquitin‐Proteasome System,” PLOS Pathogens 18, no. 8 (2022): e1010744. 35914008 10.1371/journal.ppat.1010744 PMC9371351

[188] N. Kerur , M. V. Veettil , N. Sharma‐Walia , et al., “IFI16 Acts as a Nuclear Pathogen Sensor to Induce the Inflammasome in Response to Kaposi Sarcoma‐Associated Herpesvirus Infection,” Cell Host & Microbe 9, no. 5 (2011): 363–375. 21575908 10.1016/j.chom.2011.04.008 PMC3113467

[189] V. V. Singh , N. Kerur , V. Bottero , et al., “Kaposi's Sarcoma‐Associated Herpesvirus Latency in Endothelial and B Cells Activates Gamma Interferon‐Inducible Protein 16‐Mediated Inflammasomes,” Journal of Virology 87, no. 8 (2013): 4417–4431. 23388709 10.1128/JVI.03282-12 PMC3624349

[190] D. G. Meckes, Jr. , H. P. Gunawardena , R. M. Dekroon , et al., “Modulation of B‐Cell Exosome Proteins by Gamma Herpesvirus Infection,” Proceedings of the National Academy of Sciences of the United States of America 110, no. 31 (2013): E2925–E2933. 23818640 10.1073/pnas.1303906110 PMC3732930

[191] C. Viollet , D. A. Davis , M. Reczko , et al., “Next‐Generation Sequencing Analysis Reveals Differential Expression Profiles of MiRNA‐mRNA Target Pairs in KSHV‐Infected Cells,” PLoS One 10, no. 5 (2015): e0126439. 25942495 10.1371/journal.pone.0126439 PMC4420468

[192] J. Qin , W. Li , S. J. Gao , and C. Lu , “KSHV MicroRNAs: Tricks of the Devil,” Trends in Microbiology 25, no. 8 (2017): 648–661. 28259385 10.1016/j.tim.2017.02.002 PMC6904892

[193] O. Yogev , D. Lagos , T. Enver , and C. Boshoff , “Kaposi's Sarcoma Herpesvirus MicroRNAs Induce Metabolic Transformation of Infected Cells,” PLoS Pathogens 10 (2014): e1004400. 25255370 10.1371/journal.ppat.1004400 PMC4177984

[194] O. Yogev , S. Henderson , M. J. Hayes , et al., “Herpesviruses Shape Tumour Microenvironment Through Exosomal Transfer of Viral MicroRNAs,” PLOS Pathogens 13, no. 8 (2017): e1006524. 28837697 10.1371/journal.ppat.1006524 PMC5570218

[195] P. E. Chugh , S. H. Sin , S. Ozgur , et al., “Systemically Circulating Viral and Tumor‐Derived MicroRNAs in KSHV‐Associated Malignancies,” PLoS Pathogens 9, no. 7 (2013): e1003484. 23874201 10.1371/journal.ppat.1003484 PMC3715412

[196] S. Hoshina , T. Sekizuka , M. Kataoka , et al., “Profile of Exosomal and Intracellular MicroRNA in Gamma‐Herpesvirus‐Infected Lymphoma Cell Lines,” PLoS One 11, no. 9 (2016): e0162574. 27611973 10.1371/journal.pone.0162574 PMC5017767

